# CRISPR/Cas9 Mediates Efficient Conditional Mutagenesis in *Drosophila*

**DOI:** 10.1534/g3.114.014159

**Published:** 2014-09-05

**Authors:** Zhaoyu Xue, Menghua Wu, Kejia Wen, Menda Ren, Li Long, Xuedi Zhang, Guanjun Gao

**Affiliations:** School of Life Sciences, Tsinghua University, Beijing 100084, China

**Keywords:** CRISPR/Cas9, conditional mutagenesis, gRNA, Gal4, *Drosophila*

## Abstract

Existing transgenic RNA interference (RNAi) methods greatly facilitate functional genome studies via controlled silencing of targeted mRNA in *Drosophila*. Although the RNAi approach is extremely powerful, concerns still linger about its low efficiency. Here, we developed a CRISPR/Cas9-mediated conditional mutagenesis system by combining tissue-specific expression of Cas9 driven by the Gal4/upstream activating site system with various ubiquitously expressed guide RNA transgenes to effectively inactivate gene expression in a temporally and spatially controlled manner. Furthermore, by including multiple guide RNAs in a transgenic vector to target a single gene, we achieved a high degree of gene mutagenesis in specific tissues. The CRISPR/Cas9-mediated conditional mutagenesis system provides a simple and effective tool for gene function analysis, and complements the existing RNAi approach.

Conditional mutagenesis techniques are required for analyzing the pleiotropic functions of genes at various life stages, especially for genes critical for embryogenesis. Available transgenic RNA interference (RNAi) resources in *Drosophila* are powerful tools for genomic screens and have been used for many studies of gene function (Dietzl *et al.* 2007; Ni *et al.* 2011). This method uses the Gal4-upstream activating site controlled hairpins to trigger sequence-specific mRNA breakdown. Tissue-specific gene silencing is achieved via a cross between UAS-hairpin and Gal4 driver lines; however, the RNAi method suffers from a few drawbacks. For instance, gene expression is rarely completely blocked by RNAi, frequently leading to low efficiency in gene silencing. Furthermore, for reasons unknown, some tissues such as testis and ovary (Raychaudhuri *et al.* 2012) are less sensitive to RNAi. Although one can always use the traditional approach for making conditional alleles, such as using FLP (fippase)/FRT (fippase recognition target) to generate clones when RNAi fails, the targeting procedures can be time- and labor-consuming (Choi *et al.* 2009; Gratz *et al.* 2014; Xue *et al.* 2014).

Recently, the RNA-guided CRISPR/Cas9 technology has shown potential for highly efficient genome editing in many organisms, including *Drosophila* (Bassett *et al.* 2013; Golic 2013; Gratz *et al.* 2013, 2014; Yu *et al.* 2013). Stable expression of Cas9 nuclease driven by a germline-specific promoter can induce efficient germline-transmitted mutagenesis in F_1_ progeny (Kondo and Ueda 2013; Ren *et al.* 2013; Xue *et al.* 2014). In addition, the CRISPR/Cas9 system has been successfully applied for conditional genome editing in *C. elegans*, mouse, and rat (Ma *et al.* 2013; Yang *et al.* 2013; Liu *et al.* 2014). More recently, UAS-driven expression of Cas9 in *Drosophila* has been shown to work with the CRISPR/Cas9 system (Port *et al.* 2014). Based on the high efficiency of Cas9/gRNA gene targeting, we reasoned that directly disrupting targeted genes at the DNA level would destroy gene function more efficiently than posttranscriptional breakdown of the targeted mRNA mediated by RNAi. Therefore, we combined the CRISPR/Cas9 and *Gal4-UAS* systems to develop a CRISPR/Cas9-mediated conditional mutagenesis (CMCM) method and systematically investigated whether it can efficiently inactivate gene activity in most *Drosophila* tissues. This method contains two steps (Figure 1). First, we generated gene-specific gRNAs directed against a number of genes with known phenotypes and made their 10UAS-Cas9/gRNAs transgenic flies. Second, we induced the expression of 10UAS-Cas9/gRNAs driven by the tissue-specific Gal4 lines and investigated the efficiency of gene disruption and associated phenotypes. Using the CMCM system, we tested six genes (*y*, *notch*, *bam*, *nos*, *cid*, and *ms(3)k81*) and achieved highly effective disruption in wing, eye, ovary, and testis tissues. Moreover, side-by-side comparisons of tissue-specific gene disruption showed the power of the CMCM system. In general, it was more efficient and rapid than RNAi approaches and provided a novel genetic tool for studying gene function in specific tissues *in Drosophila* (Dietzl *et al.* 2007; Ni *et al.* 2011).

**Figure 1:**
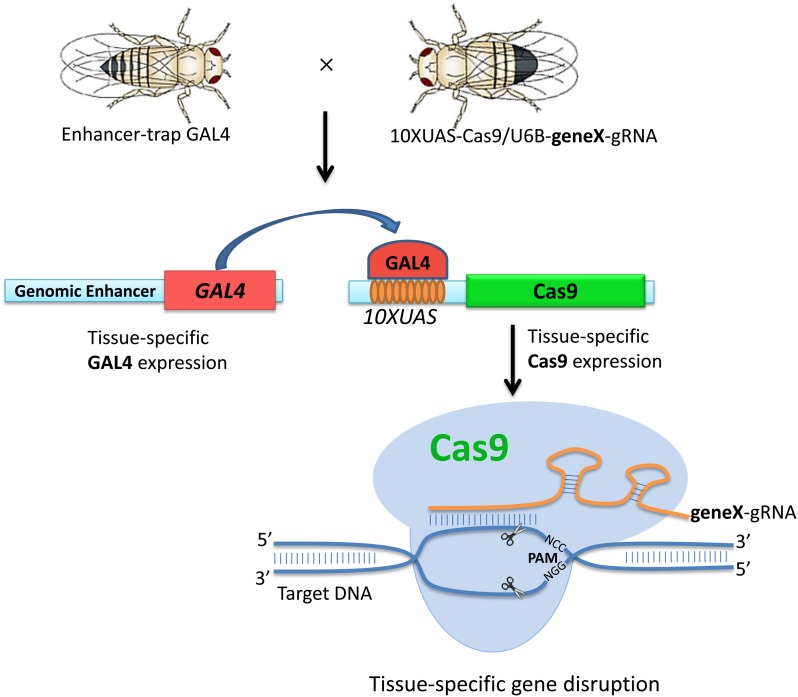
A schematic overview of the CMCM system. For each gene of interest (gene X), transgenic stocks of U6B-geneX-gRNA, which ubiquitously express gRNA targeting the gene, were established. Transgenic 10ΧUAS-Cas9/U6B-geneX-gRNA male flies were recovered after crossing the 10XUAS-Cas9 stock with the U6B-geneX-gRNA stock. The recovered male flies were crossed with the enhancer-trap Gal4 virgin flies to drive Cas9 expression in specific tissues. Mutations were induced only in the specific tissue determined by the Gal4 stocks.

## Materials and Methods

### Fly stocks

Two wild-type stocks (*w^1118^* and *y*) and four *Gal4* stocks [*tubulin(tub)-Gal4/TM6B,Tb* (Ni *et al.* 2009), *C96-Gal4* (Gustafson and Boulianne 1996), *GMR-Gal4* (Ni *et al.* 2009), and *Nos-Gal4* (Doren *et al.* 1998)] were used. Four *cid* RNAi lines were used, as follows: HMS02160 was provided by the Bloomington Stock Center (Bloomington, IN), and Vienna *Drosophila* RNAi Center (VRDC) 43856, VRDC43857, and VRDC109020 were provided by the VRDC. All balancers were obtained from the Bloomington Stock Center.

### Plasmid construction

#### PiggyBac-10UAS-Cas9 construction:

PiggyBac-10UAS-Cas9 construction required three steps. First, the 10UAS-HSP70 promoter (Supporting Information, Figure S8) was amplified from the VRDC RNAi flies by polymerase chain reaction (PCR) and was then cloned into an *Xma*I/*Not*I double-digested PiggyBac-*vasa*-cas9 plasmid (Xue *et al.* 2014) to generate PiggyBac-*10UAS*-Cas9-vasUTR. Second, an 842-bp αTub84B 3′-UTR-containing sequence (Figure S8) was released from the pM3×P3-RFPattP′ vector (Bischof *et al.* 2007) by *Xho*I/*Sal*I and then inserted into the *Sal*I-digested pSP6-2SNL-spcas9 (stored in our lab) to generate the pSP6-2SNL-spcas9-αTub84B3′-UTR. Third, the larger fragment containing cas9-αTub84B3′-UTR was cut from pSP6-2SNL-spcas9-αTub84B3′-UTR by *Not*I/AscI and cloned into the PiggyBac-10UAS-Cas9-vasUTR plasmid using the same restriction enzymes to yield the final transgenic vector PiggyBac-10UAS-Cas9 (Figure S1A).

#### pRFP-gRNA construction:

Construction of the standard entry plasmid, pRFP-gRNA, was similar to that reported for the pUAST-RFP-gRNA vector (Xue *et al.* 2014), with slight modifications (Figure S1B). To generate multiple gRNA insertions against the same target locus in the transgenic vector, pRFP-gRNA, several different subcloning vectors (TA-U6B/CR7T-gRNA-A, TA-U6B/CR7T-gRNA-B, TA-U6B/CR7T-gRNA-C, and TA-U6BCR7T-gRNA-D) harboring different enzyme restriction sites were constructed as previously described (Xue *et al.* 2014) (Figure S1C). The KOD Mutagenesis Kit (TOYOBO, Osaka, Japan) was used to insert 20-bp target sequences between the promoter and the gRNA scaffold. For single gRNA insertions, the target-specific U6B/CR7T-gRNA was released from the TA-U6B/CR7T-gRNA-A vector by digestion with *Spe*I/*Kpn*I and then cloned into pRFP-gRNA using the same sites to generate the final transgenic gene-specific vectors, pRFP-U6B/CR7T-1gRNA. For two gRNA insertions, the first gRNA was inserted into the TA-U6B/CR7T-gRNA-A plasmid (between *Spe*I and AgeI), and the second gRNA was inserted into TA-U6B/CR7T-gRNA-B (between AgeI and *Kpn*I). Next, the second insertion was released by digestion with AgeI/*Kpn*I and inserted into TA-U6B/CR7T-gRNA-A using the same restriction enzymes to generate the TA-U6B/CR7T-2gRNAs vector. Finally, the fragment containing two gRNA insertions was released from the vector TA-U6B/CR7T-2gRNAs by digestion with *Spe*I/*Kpn*I and then cloned into pRFP-gRNA to generate the final transgenic gene-specific vectors, pRFP-U6B/CR7T-2gRNAs. Using a similar method, we inserted a third and fourth gRNA directly into the pRFP-2gRNAs vector via single enzyme sites (*Spe*I and *Kpn*I, respectively) to generate transgenic gene-specific vectors pRFP-U6B/CR7T-3gRNAs and pRFP-U6B/CR7T-4gRNAs. In total, 13 transgenic pRFP-gRNAs vectors were generated. The details are presented in Table S1. All primers are listed in Table S2 and Table S3.

### Fly transformation and genetics

To obtain transgenic 10UAS-Cas9 flies, the PiggyBac-10UAS-cas9 vector was mixed with helper vector PiggyBac-transposase (Thibault *et al.* 2004) and injected into *w^1118^* fly embryos. Flies carrying 10UAS-Cas9 were identified by green fluorescent protein (GFP) expression in the eye when viewed under a fluorescence stereomicroscope. Three independent 10UAS-Cas9 lines were generated, mapping to chromosome X, chromosome 2, and chromosome 3. The 10UAS-Cas9 line mapping to chromosome 3 was used for the following experiments. To obtain transgenic gRNA flies, 13 pRFP-U6B/CR7T-gRNA vectors were separately injected into *w^1118^* fly embryos with a hyperactive *P*-element transposase (Beall *et al.* 2002), and at least two independent lines per transgenic gRNA were identified by observing the RFP eye marker. After 10UAS-Cas9 flies were crossed with the transgenic gRNA flies, F_1_ male flies expressing both GFP and RFP fluorescent markers in the eyes were collected and crossed with Gal4 virgin flies (the screen marker was red eyes). In the F_2_ progeny, flies expressing GFP, RFP fluorescent markers, and the red eye marker were the conditional mutant flies. For most gRNA transgenic vectors, we picked up just one transgene to perform subsequent crossing experiments and phenotype estimation, even if we recovered several gRNA transgenic lines. For some gRNA transgenic vectors such as CR7T-*bam*1, U6B-*nos*1*nos*2, and CR7T-*nos*1*nos*2, we picked up two independent gRNA transgenic lines for conditional mutation analyses for each transgenic vector.

### Phenotypic and genotypic analysis of conditional mutant flies

The phenotypes and genotypes of conditional mutant flies were assessed. For *yellow*, the abdomen of the conditional mutant flies was photographed with a Leica Mz10f. For *notch*, the wings of the conditional mutant flies were scored and photographed with a Leica Mz10f. For *bam* and *nos*, each of the conditional mutant female flies was crossed with three *w^1118^* male flies to test the fertility. The ovaries were scored based on their severity of phenotype and photographed with a Leica Mz10f. For *ms(3)k81* and *cid*, each conditional mutant male fly was crossed with three *w^1118^* female virgin flies to test the fertility. The testes were scored according to their fertility and photographed with a Zeiss Imager.Z2. The testes, tips of testes, seminal vesicles, and mature sperm from all conditional mutant flies and control flies were examined under a microscope with light and 4′,6-diamidino-2-phenylindole (*i.e.*, DAPI) staining. The target genomic region from the conditional mutant flies was amplified by PCR using appropriate primers following a previous protocol (Carvalho *et al.* 2009) (Table S4). For *yellow*, the whole fly was used for PCR; for *notch*, the wing, eye, and body were used for PCR; for *bam* and *nos*, the ovary was used for PCR; for *ms(3)k81* and *cid*, the testis was used for PCR. The corresponding PCR products were then sequenced.

## Results and Discussion

### Overview of the CMCM system

Transgenic Piggybac-10UAS-cas9 (Figure S1A) flies were created to express the Cas9 nuclease under control of the Gal4-driven activating site (UAS). Transgenic gRNA vectors pRFP-U6B-gRNA and pRFP-CR7T-gRNA (Xue *et al.* 2014) (modified from a pUAST plasmid with RFP replacement of the *white* gene; and deletion of the UAS site; Figure S1B) were based on two different long noncoding RNA promoters, and corresponding gRNAs were ubiquitously expressed in whole tissues. Using these vectors, we generated gene-specific gRNAs directed against genes with known phenotypes (Table S1). To increase the mutation efficiency, we designed gRNA vectors containing one to three gRNAs directed against different regions of the same gene (Figure S1C). To evaluate tissue bias with respect to the mutation efficiency of gRNA transcripts, we used both promoters to drive gene-specific gRNA transcription in specific tissues (Xue *et al.* 2014). Furthermore, to test the versatility of the CMCM method, we conducted experiments in body, wing, testis, and ovary tissues at 25° and 28°.

### CMCM-mediated conditional mutation of the *yellow* gene

**Figure 2:**
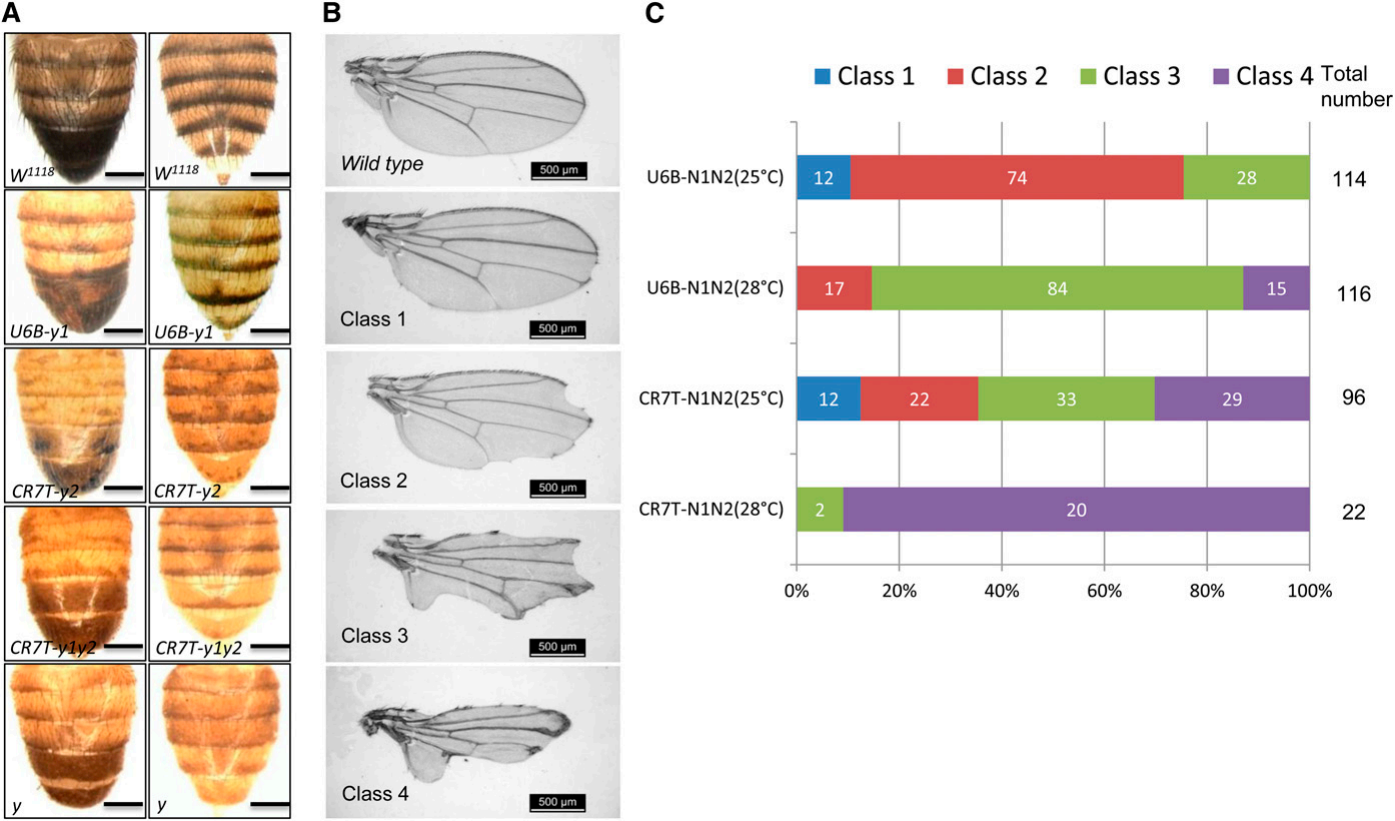
Conditional mutation of the *yellow* and *notch* genes. (A) The yellow mosaic phenotype is shown for the three different gRNA expression vectors, *U6B-y1*, *CR7T-y2*, and *CR7T-y1y2*. *Tub-Gal4/Tm6b* was used to drive the expression of Cas9. *w^1118^* and *y* flies are shown as controls. Scale bars: 200 µm. (B) Four classes of wing defects were identified when cas9 was expressed using the wing margin *C96-Gal4* driver. Class 1: margin bristles missing but no notches; Class 2: margin bristles missing and moderate wing notching; Class 3: extensive margin bristle loss and notching; and Class4: most of the wing margin missing. Scale bars: 500 µm. (C) Phenotypes of conditional mutant flies for *notch* in the wing.

To test the effectiveness of the CMCM system, we first chose the *yellow* (*y*) locus owing to its easily detectable body color phenotype. We generated transgenic fly lines containing pRFP-U6B/CR7T-gRNA constructs that target *y*. The 10UAS-Cas9 and gRNA transgenic lines were crossed to obtain 10UAS-Cas9/gRNA animals. We then induced 10UAS-Cas9 expression with *Tub-Gal4/Tm6b*, a line showing ubiquitous high-level expression of the Gal4 activator. All of the lines displayed yellow mosaicism or a completely penetrant yellow phenotype, as expected (Figure 2A), indicating that 10UAS-Cas9/gRNA triggered highly efficient gene disruption in somatic tissues. The U6B and CR7T promoters were equally efficient. The phenotype obtained with CR7T-y1y2-gRNA was indistinguishable from that of *y* mutant flies, suggesting that targeting a gene with multiple gRNAs improves the efficiency of the conditional mutagenesis. Sequencing of the *y* locus confirmed cleavage at the targeted site (Figure S2).

### CMCM-mediated conditional mutation of the *notch* gene in the wing and eye

We also tested the specificity and sensitivity of the CMCM system using the *Notch* gene, which encodes a signaling receptor essential for wing development (Johnston and Edgar 1998; Presente *et al.* 2002). We designed two gRNAs against the *Notch* gene and performed a sensitive wing assay whereby 10UAS-Cas9 was expressed under the control of the *C96-Gal4* driver, a line that expresses Gal4 in the blade region of the wing imaginal disc (Gustafson and Boulianne 1996; Presente *et al.* 2002). Disruption of *notch* led to marked defects at the wing margin ranging from mild loss of margin bristles to strong wing notching (Figure 2B), consistent with the phenotypes obtained through RNAi (Ni *et al.* 2008). We consistently observed stronger phenotypes when gRNA expression was driven by the CR7T promoter rather than by the U6B promoter, likely reflecting a difference in promoter activity in the wing (Figure 2C). We further analyzed the *notch* gene sequence in wing, eye, and body tissues of *C96-Gal4* driver-mutated flies. Mutagenesis at the designed site was observed only in the wing, not in the eye or body (Figure S3). In turn, expression of 10UAS-Cas9/gRNAs against *notch* under the control of an eye-specific *GMR-Gal4* driver generated strong defects in eye tissue, but no defects in the wing (Figure S4).

To determine how temperature affects the severity of the *notch* phenotype induced by 10UAS-Cas9/gRNA, we used the *C96-Gal4* driver at both 25° and 28°. Phenotypes obtained were stronger at 28° than at 25° for both promoters. A large fraction of flies (20 of 22) showed extreme defects (class 4) at 28° when using the CR7T promoter, indicating an effect of temperature.

### CMCM-mediated conditional mutations of the *bam* and *nos* genes in the ovary

**Figure 3:**
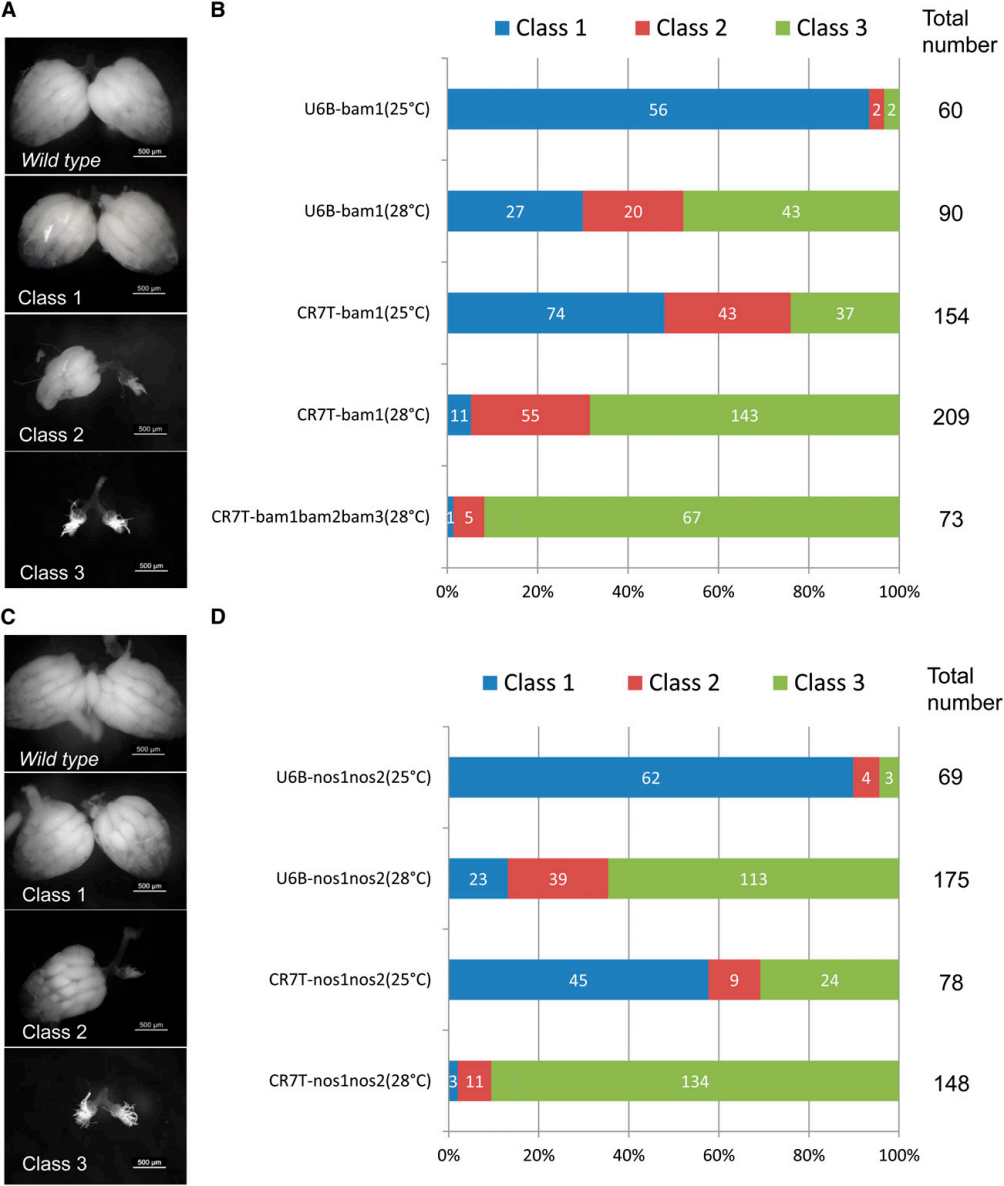
Conditional mutations of the *bam* and *nos* genes in the ovary. (A, C) Three classes of ovary defects were distinguished when 10UAS-cas9 was expressed using the *nos-Gal4* driver for both *bam* and *nos* genes. Class 1: the same as wild-type; Class 2: one side of the ovary was shrunken and the other side was normal; and Class 3: both sides of the ovary were shrunken. (B, D) Phenotypes of the *bam* and *nos* genes in the ovaries of conditional mutant flies. High temperature and the use of multiple gRNAs can improve the efficiency of CMCM, and the CR7T promoter works better than the U6B promoter in the ovary.

*Drosophila* oogenesis is an important model system in developmental and evolutionary biology. We next applied CMCM to the ovary using the *bam* (*bag of marbles*) gene. Genetic *bam*-mutants and *bam*-RNAi flies showed disrupted cyst formation during oogenesis, resulting in ovaries containing an excess number of cells that cannot differentiate into gametes (McKearin and Spradling 1990; Chen and McKearin 2003; Ni *et al.* 2011). We induced 10UAS-Cas9 expression in the ovary using *nos-Gal4*, a line expressing Gal4 in all stages of oogenesis (Rørth 1998). We observed phenotypes consistent with genetic mutants and RNAi flies (Figure 3A). A larger fraction of ovaries showed extreme phenotypes (class 3) when gRNA expression was driven by CR7T rather than by the U6B promoter at both 25° and 28° (Figure 3B). Furthermore, disruption of *bam* driven by multiple gRNAs led to extreme phenotypes in more than 90% of females (class 3) at 28° (Figure 3B). Disruption of *nos* driven by *nos-Gal4* caused a phenotype similar to that resulting from *nos-Gal4*-driven *bam* disruption (Figure 3, C and D). Sequencing of dissected mutated ovaries showed many mutations at the designed targeting site (Figure S5 and Figure S6).

### CMCM-mediated conditional mutations of the *cid* and *ms(3)k81* genes in the testis

**Figure 4:**
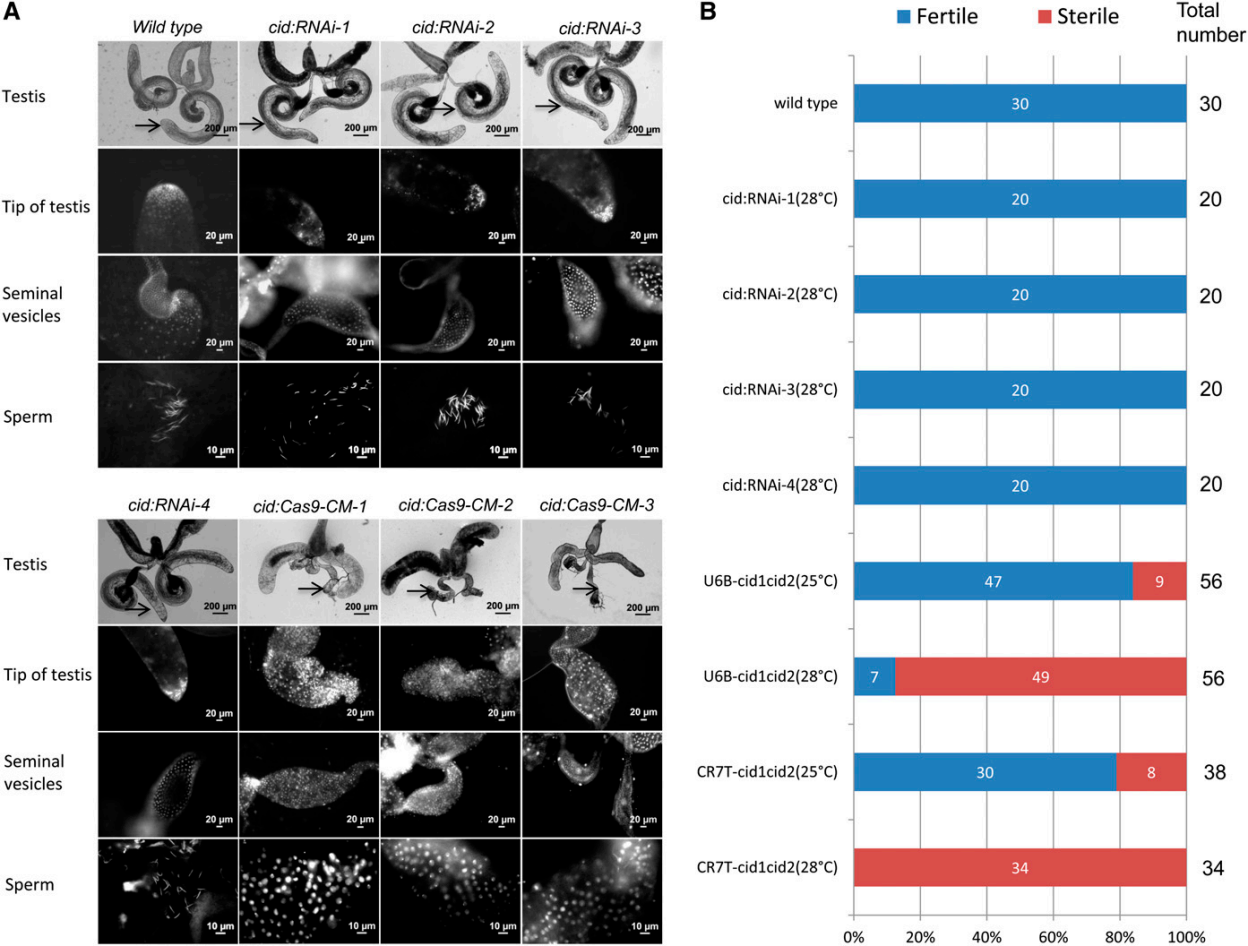
Conditional mutation of the *cid* gene in the testis. (A) The phenotypes in the testis tissue are compared for the RNAi lines and CMCM lines. From top to bottom of each column: whole testis (light), tip of the testis (DAPI), seminal vesicles (DAPI), and sperm detection (DAPI). The arrows indicate testis tissue. *cid:RNAi-1*, BM40912; *cid:RNAi-2*, v43856; *cid:RNAi-3*, v43857; *cid:RNAi-4*, v109020. *cid:Cas9-CM* denotes testis from a conditional mutant fly via the CMCM system; *nos-Gal4* was used to drive the expression of Cas9, and two vectors (*U6B-cid1cid2* and *CR7T-cid1cid2*) were used to drive the expression of gRNA. *cid:cas9-CM-1* is from *U6B-cid1cid2 (28°C)*; *cid:cas9-CM*-2 is from *CR7T-cid1cid2 (25°C)*; *cid:cas9-CM-3* is from *CR7T-cid1cid2 (28°C)*. All examined *cid:Cas9-CM* testes lacked mature sperm in the seminal vesicles. (B) Fertility tests of flies bearing the transgenic RNAi and the CMCM system of the *cid* gene. DAPI, 4′,6-diamidino-2-phenylindole.

We also targeted the *cid* (*cenH3*) gene, which encodes a protein essential for somatic centromere assembly and embryogenesis (Blower and Karpen 2001; Blower *et al.* 2006). We induced 10UAS-Cas9 expression using the *nos-Gal4* driver to detect phenotypes in the testis. Testis size was greatly reduced (Figure 4A), probably because *nos-Gal4* is expressed very early during spermatogenesis (Rørth 1998). Disruption of the *cid* gene during early spermatogenesis led to a failure in cell division and to male sterility. We could not detect any mature sperm in the seminal vesicles of the mutated testes (Figure 4A), likely explaining the observed sterility. Low temperature−induced (25°) conditional mutagenesis using both promoters resulted in a small fraction of flies with defects in testis tissue (Figure 4B). However, induced mutagenesis at 28° resulted in a high fraction of flies with defects for both promoters. Notably, all examined mutated male flies under the control of the CR7T promoter (34 of 34) were 100% sterile and showed severe phenotypes in the testes at 28° (Figure 4B).

To directly compare the CMCM system with RNAi, we tested all four *cid*-RNAi lines from the Vienna *Drosophila* RNAi Center and Bloomington Stock Center. After crossing each of them to the *nos-Gal4* driver, all testes from RNAi flies were indistinguishable from those from wild-type flies (Figure 4A). Indeed, all examined RNAi flies (more than 20 for each line) were fertile (Figure 4B), indicating that RNAi cannot completely block the expression of *Cid* in testis tissue. A previous study also demonstrated that an RNAi-induced reduction in Cid expression during spermatogenesis has no effect on male fertility (Raychaudhuri *et al.* 2012).

To rule out any nonspecific effects of the CMCM system in testis, we next tested the male-specific gene *ms(3)k81*. Mutations in *ms(3)k81* cause male sterility without morphologically detectable defects in testis and sperm development (Fuyama 1986; Yu *et al.* 2013). We then induced 10UAS-Cas9 expression in testis under the *nos-Gal4* driver. Testis and sperm development were normal (Figure S7A). Almost 80% of CMCM-induced flies (30 of 38) were completely sterile at 28° (Figure S7B). Sequencing of these sterile flies confirmed disruption of *ms(3)K81* in testis tissue (Figure S7, C and D).

In contrast to the RNAi strategy of knocking down gene expression at the mRNA level, the CMCM approach silences a gene at the DNA level in a more complete way. For six well-known genes, we achieved very efficient gene disruption in wing, eye, ovary, and testis tissues using CRISPR/Cas9 combined with the *Gal4-UAS* system in *Drosophila*. Detailed examinations of phenotypes resulting from *notch* gene disruption in the wing and eye using tissue-specific Gal4 drivers revealed that CMCM-mediated gene disruption appears to be strictly restricted to the particular tissue.

However, the efficiency of CMCM-induced phenotypes could be affected by the following parameters: (1) the promoter of gRNA expression, (2) the choice and the number of gRNAs, (3) temperature, (4) incomplete mutation of the target gene, and (5) positional effects of randomly integrated gRNA transgenes. In this report, CR7T promoter-driven gRNAs led to more efficient conditional mutagenesis than U6B promoter-driven gRNAs in most tissues, particularly in the ovary, indicating that the pleiotropic functions of one particular gene in various tissues could be analyzed by crossing the same transgenic 10UAS-Cas9/gRNA under the CR7T promoter with different tissue-specific Gal4 drivers. Nevertheless, RNAi-mediated knockdown efficiency usually is affected by the structure and direction of hairpin RNAs, which can result in unstable mutagenesis in different tissues (Ni *et al.* 2011). To achieve the strong phenotype of gene silencing, one frequently needs to generate different hairpin RNAi lines to trigger silencing of the same gene in multiple tissue types, especially during oogenesis (Ni *et al.* 2011). Regarding the choice and the number of gRNAs, the mutation efficiency of the CMCM system depends on the gene locus. To obtain the strongest loss-of-function phenotypes, the use of multiple gRNAs against the same target site could be considered. The presence of both mutant and wild-type alleles from incomplete cleavage by Cas9 may lead to complex phenotypes. The phenotypes generated at 28° were more severe than those generated at 25°, which is consistent with the temperature sensitivity of the *Gal4-UAS* system. In addition, we did not observe significant efficiency differences in the tissue-specific mutagenesis between two independent gRNA transgenic lines from the same transgenic gRNA. Although the expression of randomly integrated gRNA is known to be influenced by their local genomic environment, position effects of transgenic gRNA in this study did not cause severe effects on the mutant phenotypes. Based on current examples, we propose that a small amount of transgenic gRNA expression may be enough for efficient tissue-specific mutagenesis. However, future CMCM-mediated conditional experiments could be performed at the fitted transgenic gRNA loci via the phiC31-mediated *attP/attB* system (Ni *et al.* 2008).

CMCM system has two important applications. First, the CR7T promoter can be used to drive multiple gRNAs to target a single gene to achieve high degree of inactivation in specific tissues; all gRNAs could easily be integrated into one simple transgenic vector using our method. Second, it provides an independent method for validation of the loss-of-function phenotypes after RNAi genetic screening. In summary, our CMCM system provides a simple and efficient strategy for conditional gene disruption, making it possible to investigate the different functions of a single gene in various tissues during development.

## Supplementary Material

Supporting Information
