# Supplementary material for: CRISPR/Cas9 Mediates Efficient Conditional Mutagenesis in Drosophila
Source: G3 (Bethesda). 2014 Sep 5;4(11):2167–73. doi: 10.1534/g3.114.014159 (PMC4232542; doi:10.1534/g3.114.014159)
Supplement: Supporting Information [file supp_4_11_2167__index.html]

CRISPR/Cas9 Mediates Efficient Conditional Mutagenesis in Drosophila — CRISPR/Cas9 Mediates Efficient Conditional Mutagenesis in Drosophila — Supporting Information 

# CRISPR/Cas9 Mediates Efficient Conditional Mutagenesis in *Drosophila*

## Supporting Information for Xue *et al.*, 2014

**Files in this Data Supplement:**

- Supporting Information - Figures S1-S8 and Tables S1-S4 (PDF, 932 KB)
- Figure S1 - Maps of the plasmids. (PDF, 421 KB)
- Figure S2 - Sequence results for the *y* conditional mutant flies. (PDF, 225 KB)
- Figure S3 - Sequence results for *notch* conditional mutant flies. (PDF, 219 KB)
- Figure S4 - Phenotypes resulting from the conditional *notch* mutation in the eye and wing. (PDF, 329 KB)
- Figure S5 - Sequence results for the ovaries of *bam* conditional mutant flies. (PDF, 230 KB)
- Figure S6 - Sequence results for the ovaries of *nos* conditional mutant flies. (PDF, 236 KB)
- Figure S7 - Conditional mutation of the *ms(3)k81* gene via the Cas9-mediated conditional mutagenesis (CMCM) system. (PDF, 331 KB)
- Figure S8 - Sequences of the 10UAS-HSP70 promoter and αTub84B 3'-UTR used in this study. (PDF, 200 KB)
- Table S1 - Sites targeted for each *Drosophila* gene and pRFP-gRNA constructions for the target loci. (PDF, 221 KB)
- Table S2 - List of primers used to construct the 10UAS-Cas9/TA-gRNA vector. (PDF, 174 KB)
- Table S3 - List of primers used to construct the transgenic gRNA vector. (PDF, 205 KB)
- Table S4 - List of primers used for PCR to verify the conditional mutations. (PDF, 203 KB)
