# Supplementary material for: CRISPR/Cas9 Mediates Efficient Conditional Mutagenesis in Drosophila
Source: G3 (Bethesda). 2014 Sep 5;4(11):2167–73. doi: 10.1534/g3.114.014159 (PMC4232542; doi:10.1534/g3.114.014159)
Supplement: Supporting Information [file supp_g3.114.014159_014159SI.pdf]

G3 · Genes | Genomes | Genetics

CRISPR/Cas9 mediates efficient conditional mutagenesis in *Drosophila*

Zhaoyu Xue[1], Menghua Wu[1], Kejia Wen[1], Menda Ren[1], Li Long[1], Xuedi Zhang[1] and Guanjun Gao[1,*]

[1]School of Life Sciences, Tsinghua University, Beijing 100084, China

*Corresponding author:

Guanjun Gao, School of Life Sciences, Tsinghua University, Yuanmingyuan Road 1, Beijing 100084, China. E-mail:

gaogu@mail.tsinghua.edu.cn

DOI: 10.1534/g3.114.014159

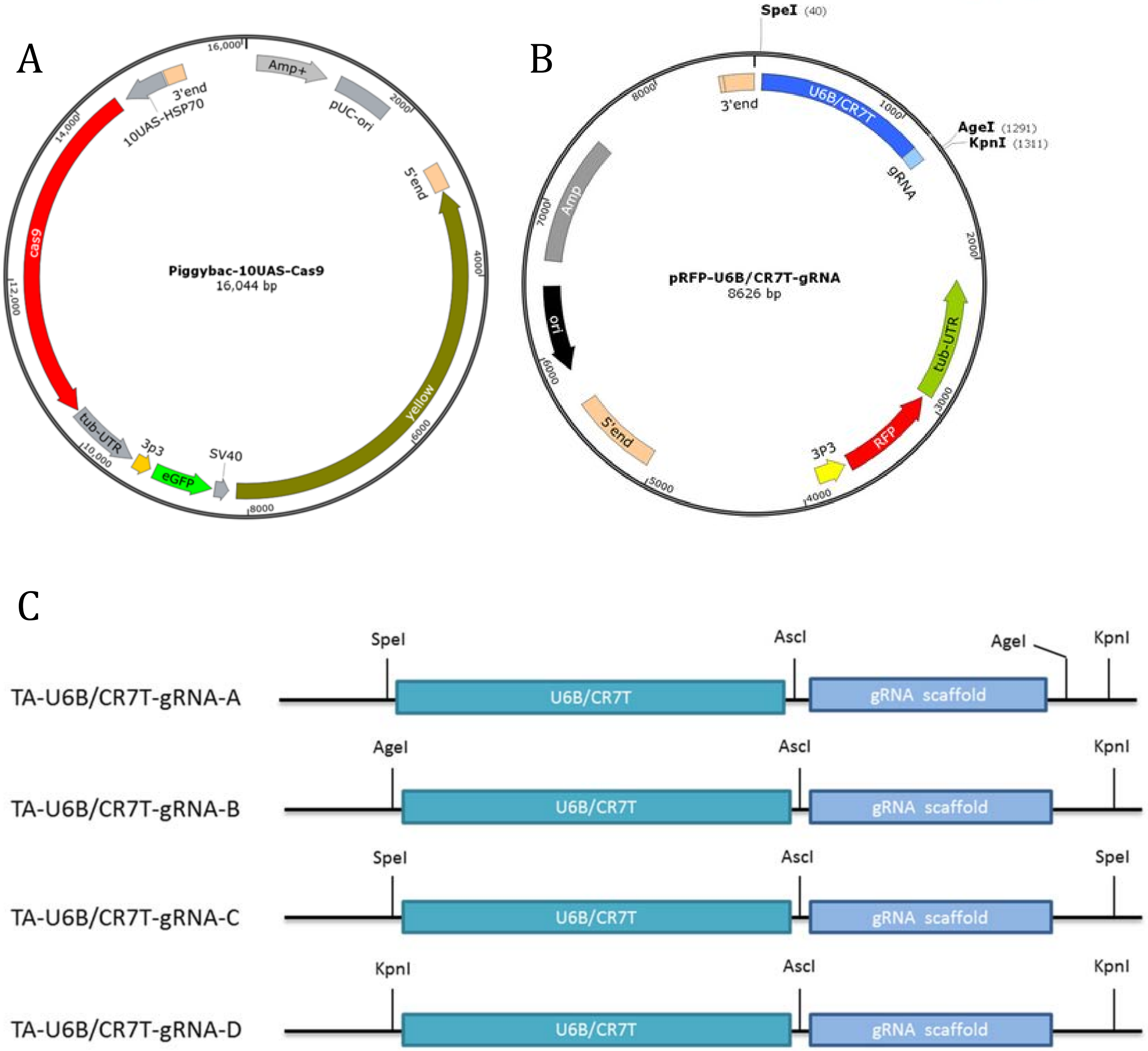
A
Piggybac-10UAS-Cas9
16,044 bp
Amp+
pUC-ori
5'end
yellow
SV40
eGFP
3p3
tub-UTR
cas9
10UAS-HSP70
3'end
16,000
2000
4000
6000
8000
10,000
12,000
14,000
B
pRFP-U6B/CR7T-gRNA
8626 bp
SpeI (40)
AgeI (1291)
KpnI (1311)
U6B/CR7T
gRNA
3'end
Amp
ori
5'end
3P3
RFP
tub-UTR
1000
2000
3000
4000
5000
6000
7000
8000
C
TA-U6B/CR7T-gRNA-A
SpeI
AscI
AgeI
KpnI
U6B/CR7T
gRNA scaffold
TA-U6B/CR7T-gRNA-B
AgeI
AscI
KpnI
U6B/CR7T
gRNA scaffold
TA-U6B/CR7T-gRNA-C
SpeI
AscI
SpeI
U6B/CR7T
gRNA scaffold
TA-U6B/CR7T-gRNA-D
KpnI
AscI
KpnI
U6B/CR7T
gRNA scaffold

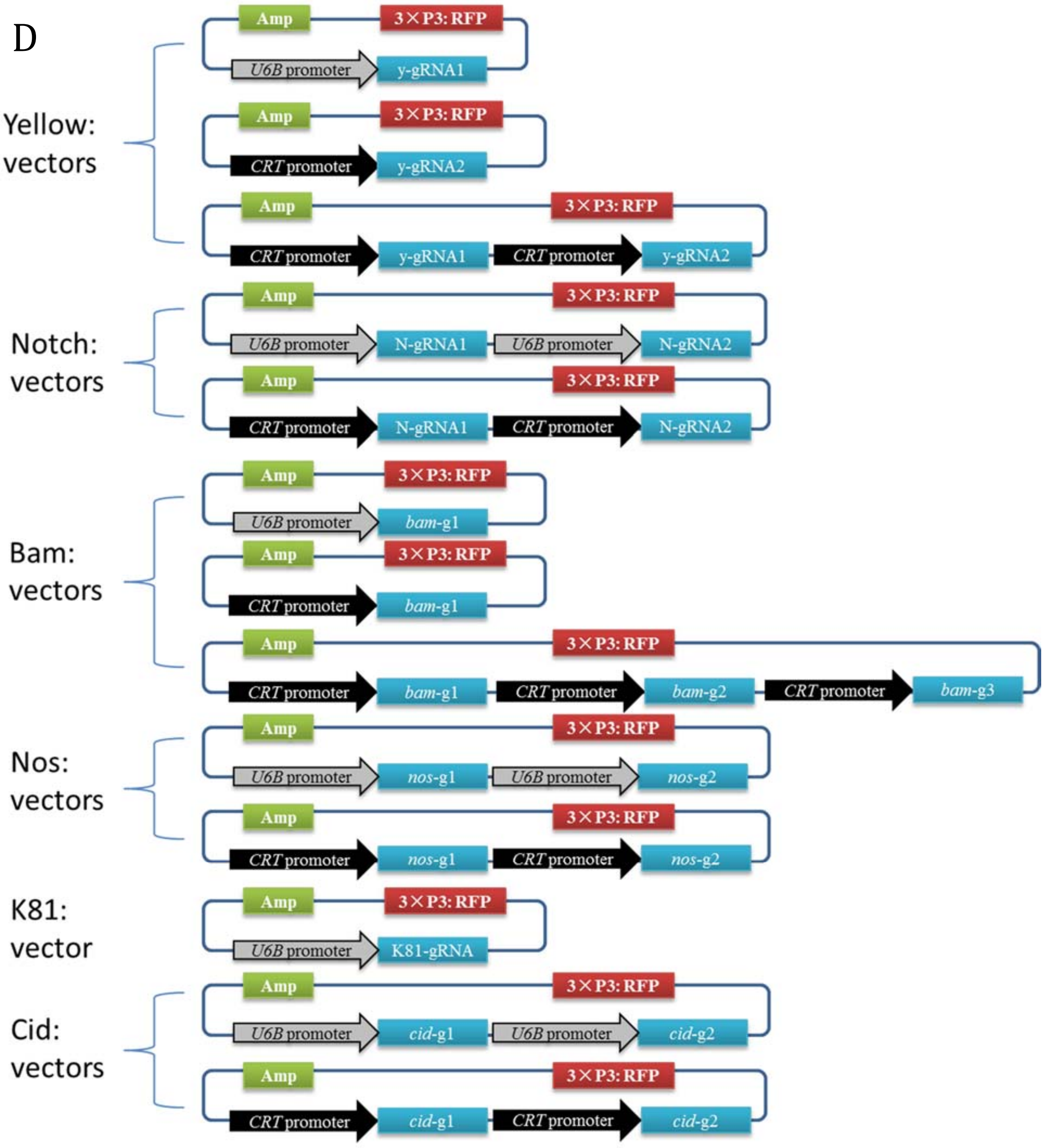

Figure S1 Maps of the plasmids. (A) Piggybac-10UAS-cas9, (B) pRFP-U6B/CR7T-gRNA, and (C) the four backbone plasmids for gRNA insertion. (D) Maps of all transgenic gRNA vectors.

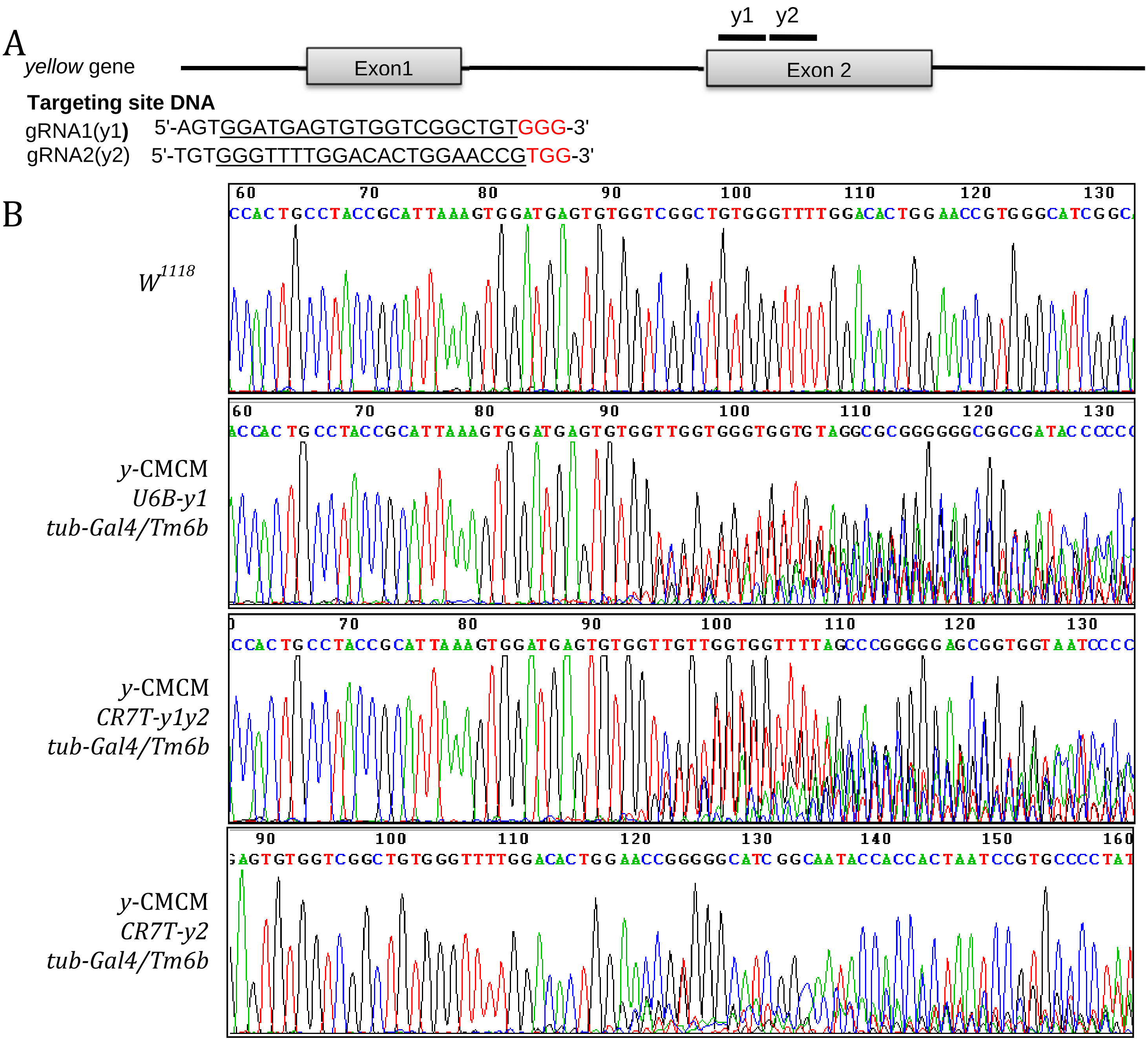

Figure S2 Sequence results for the *y* conditional mutant flies. (A) Sequences and schematic representation of the two gRNAs against the *y* gene. (B) *Tub-Gal4/Tm6b* was used to drive the expression of Cas9 in whole bodies, and three vectors, *U6B-y1*, *CR7T-y2*, and *CR7T-y1y2,* were used to drive the expression of gRNA. The mutations were induced exactly at the target locus. w^{1118} was used as the control.

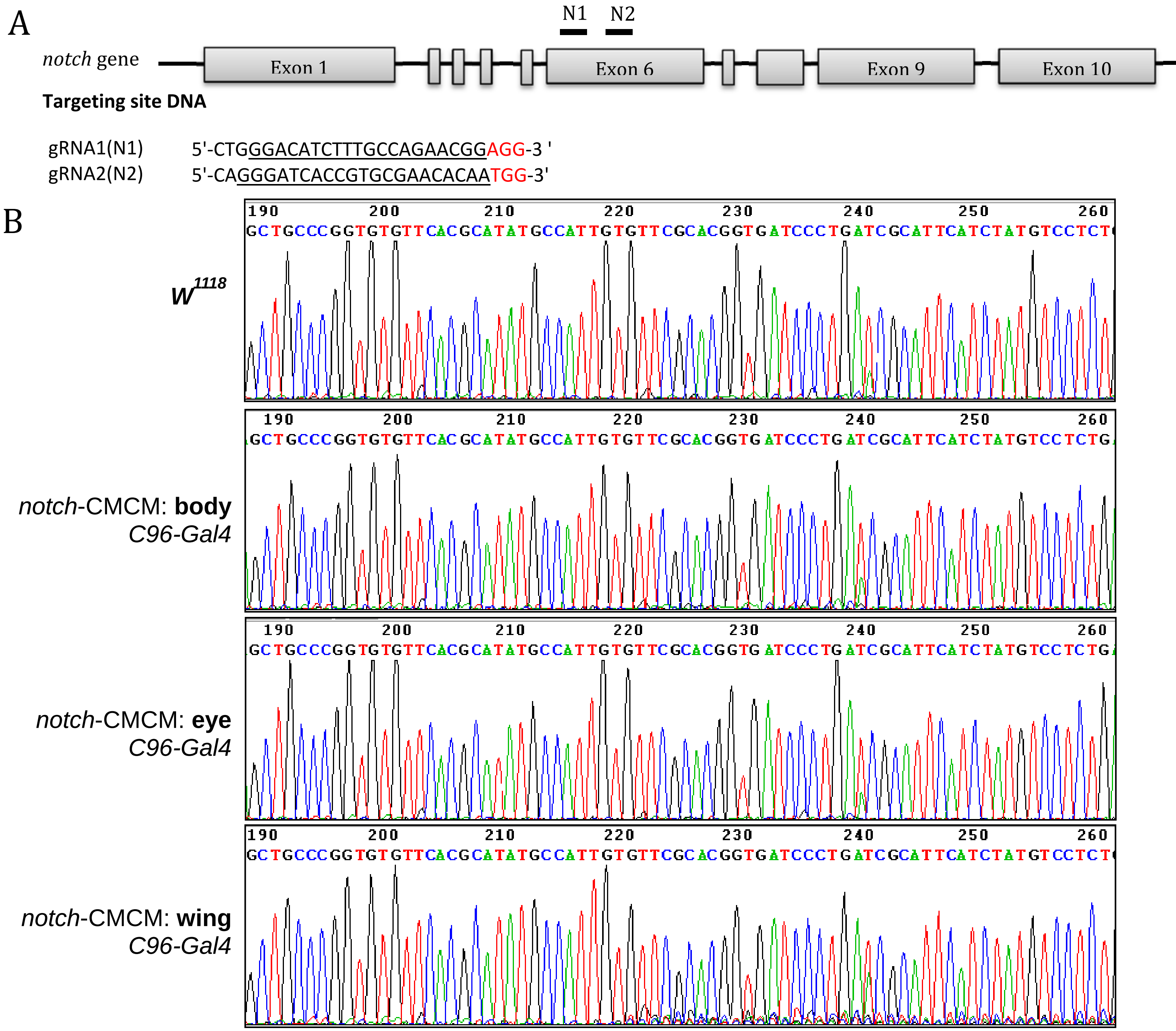

Figure S3 Sequence results for *notch* conditional mutant flies. (A) Sequences and schematic representation of two gRNAs against the *notch* gene. (B) *C96-Gal4* was used to drive the expression of Cas9 specifically in the blade region of the wing imaginal disc. The body, eye, and wing from the *notch* conditional mutant flies were sequenced. The mutation was induced only in the wing tissue at the target locus. w^{1118} was used as the control.

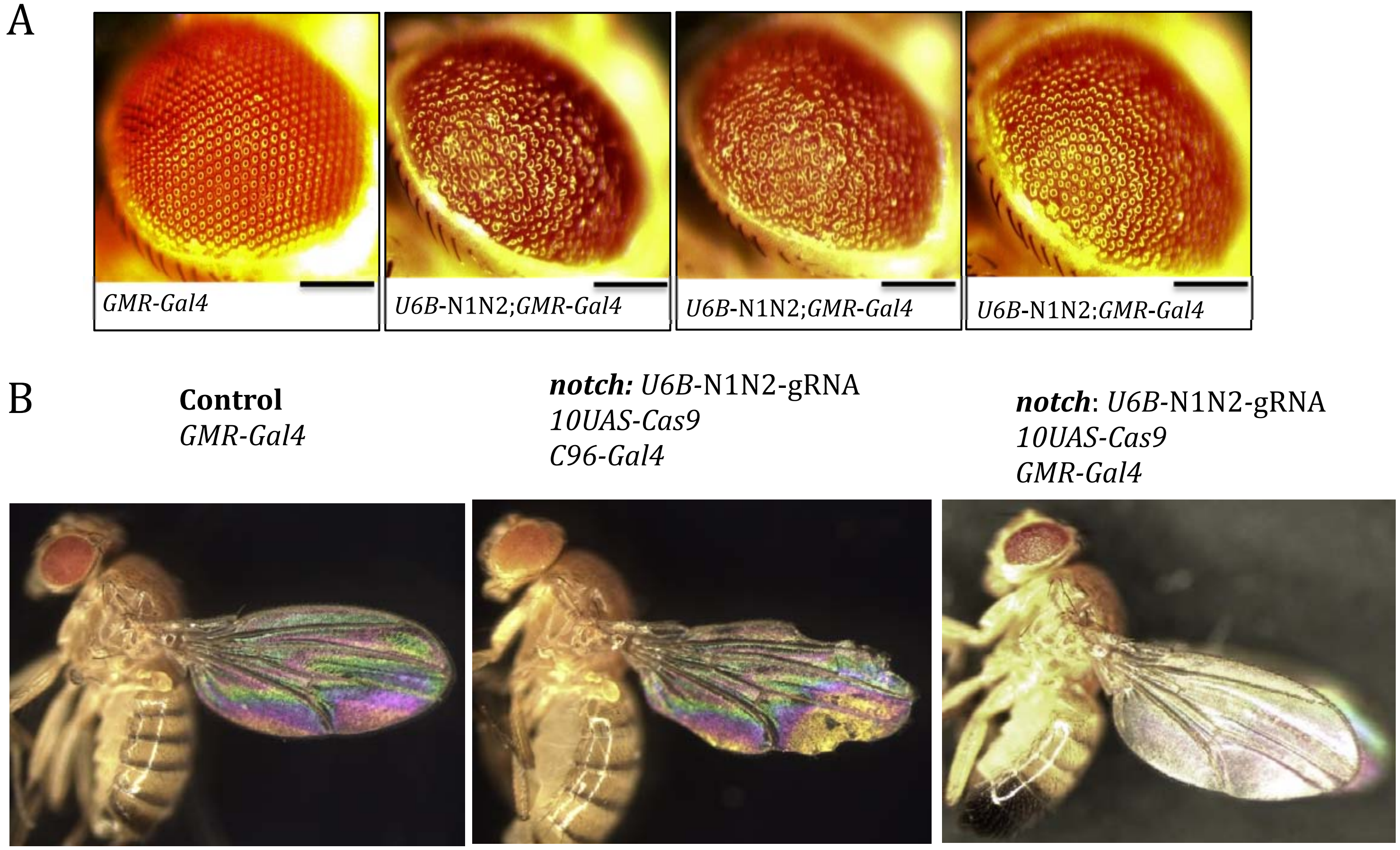

Figure S4 Phenotypes resulting from the conditional *notch* mutation in the eye and wing. (A) The conditional mutant flies showed rougher eyes than the *GMR-Gal4* control. Scale bars: 100 µm. (B) The whole-fly images for *notch* conditional mutagenesis are shown. The left-hand image used the *GMR-Gal4* fly as a control, the middle image is from the *notch* conditional mutagenesis driven by wing-specific *C96-Gal4*, and the right-hand image is from the *notch* conditional mutagenesis driven by eye-specific *GMR-Gal4*. *U6B-N1N1* was used to drive the expression of gRNA.

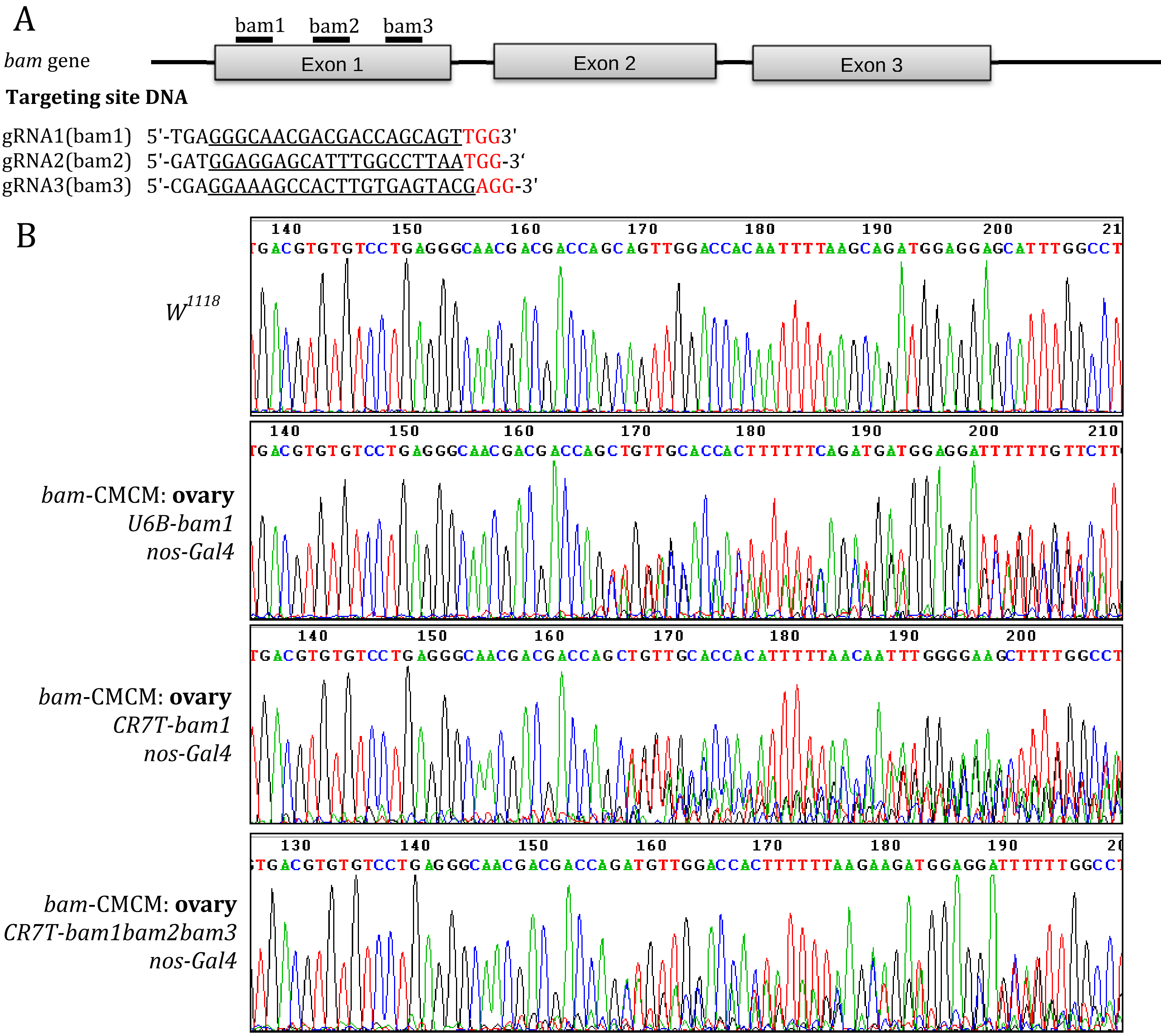

Figure S5 Sequence results for the ovaries of *bam* conditional mutant flies. (a) The sequences and a schematic representation of three gRNAs against the *bam* gene are shown. (b) *Nos-Gal4* was used to drive the expression of Cas9 specifically in the ovary, and three vectors, *U6B-bam1*, *CR7T-bam2,* and *CR7T-bam1bam2bam3*, were used to drive the expression of gRNA. The mutations were induced exactly at the target locus. A w^{1118} fly was used as the control.

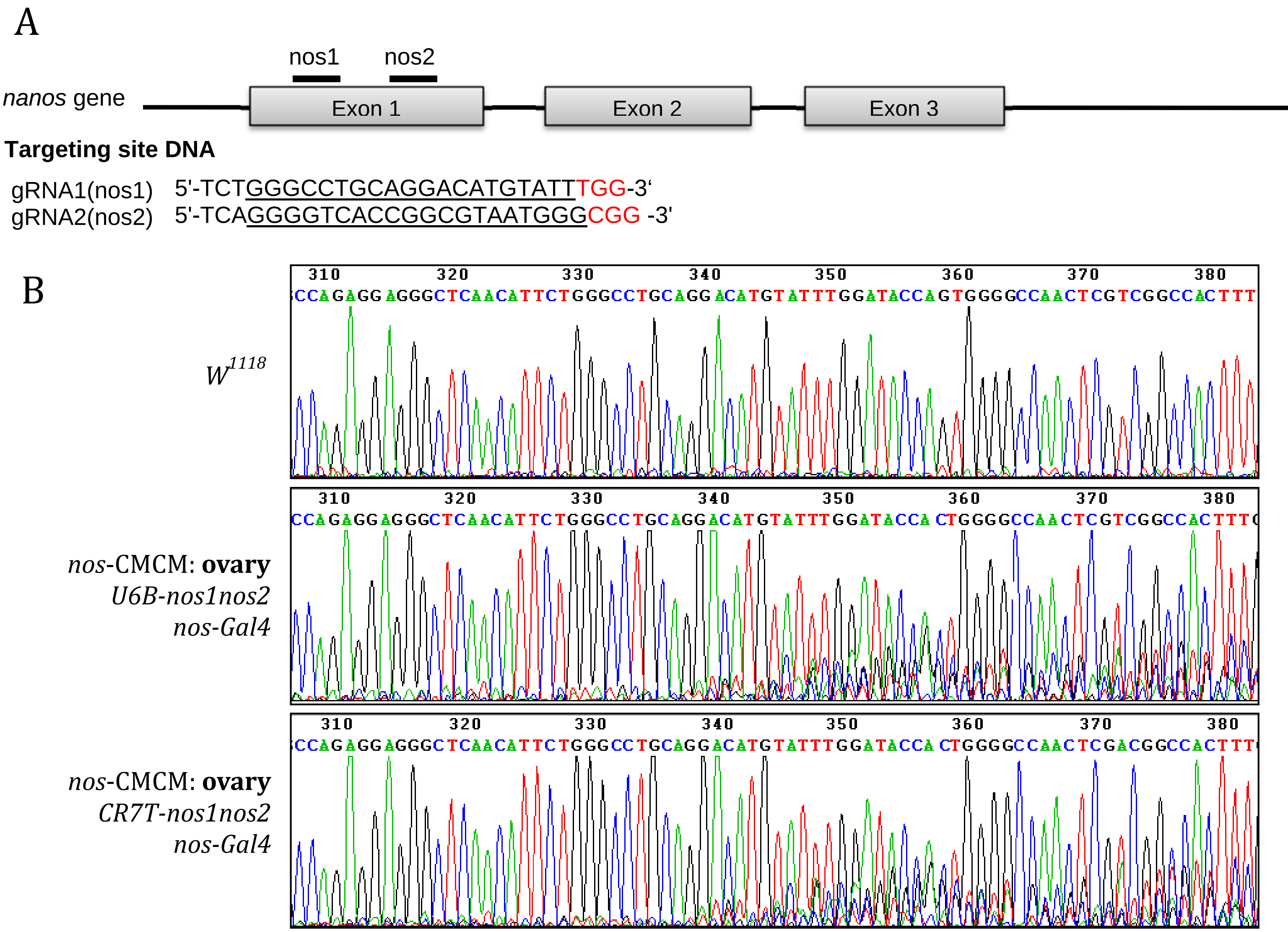

Figure S6 Sequence results for the ovaries of *nos* conditional mutant flies. (a) The sequence and a schematic representation of two gRNAs for the *nos* gene are shown. (B) *Nos-Gal4* was used to drive the expression of Cas9 specifically in the ovary, and two vectors, *U6B-nos1nos2* and *CR7T-nos1nos2*, were used to drive the expression of gRNA. The mutations were induced exactly at the target locus. A w^{1118} fly was used as the control.

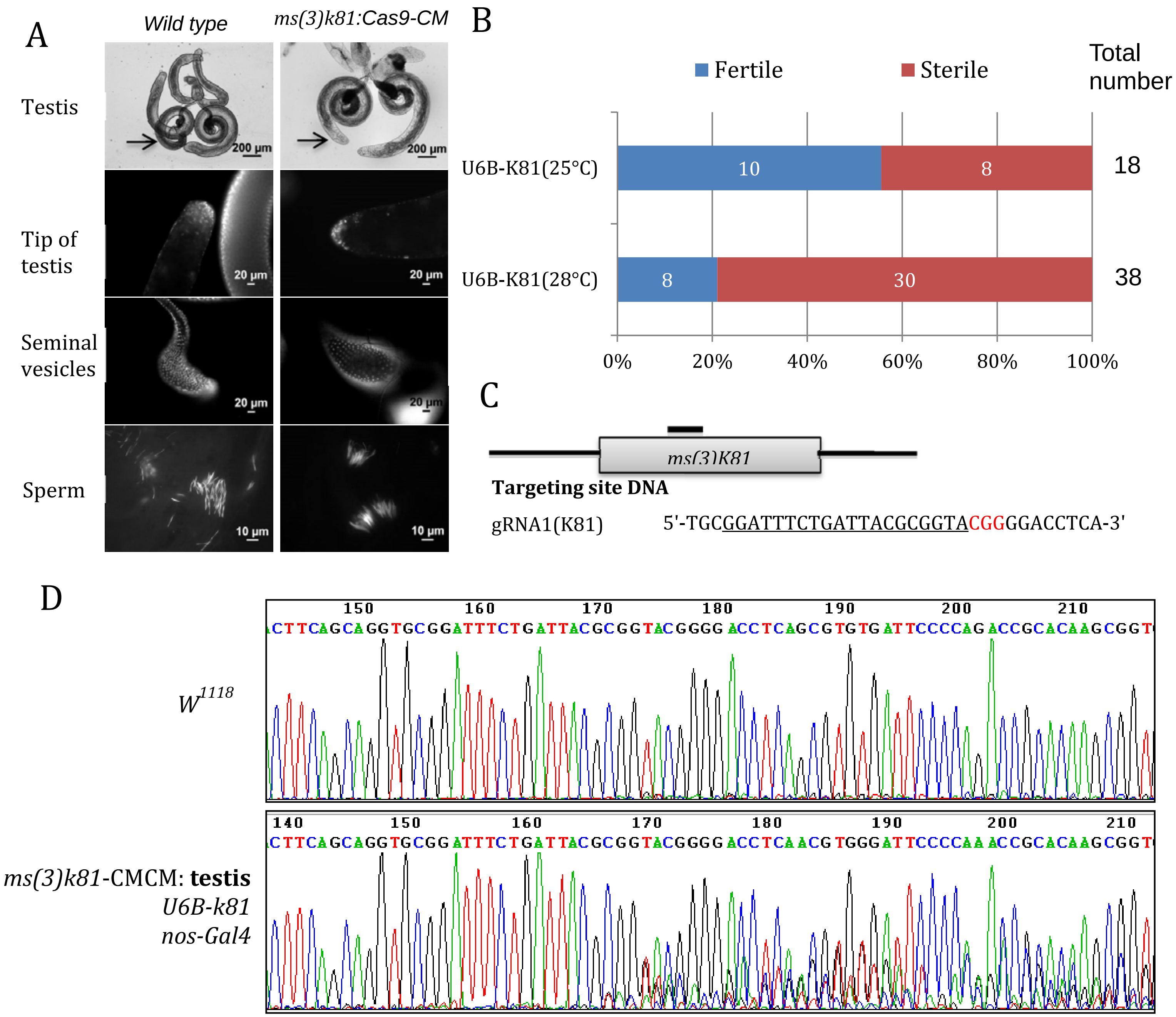

Figure S7 Conditional mutation of the *ms(3)k81* gene via the Cas9-mediated conditional mutagenesis (CMCM) system. (A) From the top to bottom of each column: whole testis (light), tip of the testis (DAPI), seminal vesicles (DAPI), and sperm detection (DAPI). *Nos-Gal4* was used to drive the expression of Cas9, and *U6B-K81* was used to drive the expression of gRNA. *ms(3)k81:Cas9-CM* was the testis from conditional mutant fly via the CMCM system. (B) Results of a fertility test are shown for flies for the CMCM system using the *ms(3)k81* gene. (C, D) The sequences and

a schematic representation of gRNAs against the *ms(3)k81* gene. Sequence results for the testes from the *ms(3)k81* conditional mutant flies. The mutations induced by CMCM are located at the targeted locus.

>10UAS-HSP70 promoter
GGCTCGATCCGCTTGCATGCCTGCAGGTCGGAGTACTGTCCTCCGAGCGGAGTACTGTCCTCC
GAGCGGAGTACTGTCCTCCGAGCGGAGTACTGTCCTCCGAGCGGAGTACTGTCCTCCGAGCGG
AAGCTTGCATGCCTGCAGGTCGGAGTACTGTCCTCCGAGCGGAGTACTGTCCTCCGAGCGGAG
TACTGTCCTCCGAGCGGAGTACTGTCCTCCGAGCGGAGTACTGTCCTCCGAGCGGAGACTCTA
GCGAGCGCCGGAGTATAAATAGAGGCGCTTCGTCTACGGAGCGACAATTCAATTCAAACAAG
CAAAGTGAACACGTCGCTAAGCGAAAGCTAAGCAAATAAACAAGCGCAGCTGAACAAGCTA
AACAATCTGCAGTAAAGTGCAAGTTAAAGTGAATCAATTAAAAGTAACCAGCAACCAAGTAA
ATCAACTGCAACTACTGAAATCTGCCAAGAAGTAATTATTGAATACAAGAAGAGAACTCTGA
ATAGGGAATTGG
>αTub84B 3’-UTR
CGGCCATCGAATTCGAGCTCGCCCACTAAGCGTCGCGCCACTTCAACGCTCGATGGGAGCGTC
ATTGGTGGGCGGGGTAACCGTCGAAATCAGTGTTTACGCTTCCAATCGCAACAAAAAATTCAC
TGCAACACTGAAAAGCATACGAAAACGATGAAGATTGTACGAGAAACCATAAAGTATTTTAT
CCACAAAGACACGTATAGCAGAAAAGCCAAGTTAACTCGGCGATAAGTTGTGTACACAAGAA
TAAAATCGGCCAGATTCAGTGTTGTCAGAAATAAGAAAACCCCACTATGTTTTTCTTTGCCTTT
TCTTTCTCCCAGCGATCATTCATTTCGTGGTGAAAGAACGGGGTCATTGCACGGAGTTTCGACT
GCGGGAAAGCAGAGCTGCCGTTCACTTCGTCTATAATTAGCGCTTTCTATTTTCCCCGATTCGG
GCCGCTGCTGCGCTTTTCCGCCTGCTGTTTGTGGCAAGTGTAGCAGCAGGCTGTGCACGCAGT
GTGGCATGCACTTGGCTTTCCACCGTTGGTATCGATTCTCTGGGACGATGAGTCATTCCTTTCG
GGGCCACAGCATAATCGTTGCCAGCTCACCGAAATGGTGACTTCATTTCTTAACTGCCGTCAA
GCATGCGATTGTACATACATACATATTTATATATGTACATATTTATGTGACTATGGTAGGTCGA
TATAATAGCAATCAACGCAAGCAAATGTGTCAGTCCTGCTTACAGGAACGATTCTATTTAGTA
ATTTTCGTTGTATAAAGTAATTATGTATGTATGTAAGCCCCATAAATCTGAAACAATTAGGCA
AAACCATGCGAAGCTCTCTA

Figure S8 Sequences of the 10UAS-HSP70 promoter and αTub84B 3’-UTR used in this study.

Table S1 Sites targeted for each *Drosophila* gene and pRFP-gRNA constructions for the target loci.

Target gene		Target site (5' to 3') (PAM is underlined)	pRFP-gRNA	Number of gRNA
yellow	y1	GGATGAGTGTGGTCGGCTGTGGG	pRFP-U6B-y1-gRNA	1
	y2	GGGTTTTGGACACTGGAACCGTGG	pRFP-CR7T-y2-gRNA	1
			pRFP-CR7T-y1y2-gRNA	2
notch	N1	GGACATCTTTGCCAGAACGGAGG	pRFP-U6B-N1N2-gRNA	2
	N2	GGGATCACCGTGCGAACACAATGG	pRFP-CRT-N1N2-gRNA	2
bag of marbles (bam)	bam1	GGGCAACGACGACCAGCAGTTGG	pRFP-U6B-bam1-gRNA	1
	bam2	GGAGGAGCATTTGGCCTTAATGG	pRFP-CR7T-bam1-gRNA	1
	bam3	GGAAAGCCACTTGTGAGTACGAGG	pRFP-CR7T-bam1bam2bam3-gRNA	3
nanos	nos1	GGGCCTGCAGGACATGTATTTGG	pRFP-U6B-nos1nos2-gRNA	2
	nos2	GGGGTCACCGGCGTAATGGGCGG	pRFP-CR7T-nos1nos2-gRNA	2
cid	cid1	GGACGCCGGACGGAGGCAGCCGG	pRFP-U6B-cid1cid2-gRNA	2
	cid2	GGAAAGCAAAACGCGAGCAGCAGG	pRFP-CR7T-cid1cid2-gRNA	2
ms(3)K81	K81	GGATTTCTGATTACGCGGTACGG	pRFP-U6B-K81-gRNA	1

Table S2 List of primers used to construct the 10UAS-Cas9/TA-gRNA vector.

Plasmid	Primer name	Primer sequence (5' – 3') Forward and Reverse
piggyBac-10UAS-cas9	10UAS-XmaI-F	CCCGGGCTCGATCCGCTTGCATGC
	10UAS-NotI-R	GCGGCCGCCAATTCCCTATTCAGAGTTCTCTTCTT
	αTub84B 3'UTR-XhoI-F	CTACTACTACTCGAGCGGCCATCGAATTCGAGCTC
	αTub84B 3'UTR-SpeISalI-R	CATCATCATGTCGACACTAGTTAGAGAGCTTCGCATGGTTTTGCC
TA-U6B/CR7T-gRNA -A	U6B-NotIsphIspeIFseI-F	GCGGCCGCATGCACTAGTGGCCGGCCGTTCGACTTGCAGCCTGAAATAC
	CR7T-XhoIspeIFseI-F	CTCGAGACTAGTGGCCGGCCCGTTTTGTCATCGCTTTTTGTCG
	U6B/CR7T-AgeI/KpnI-R	GGTACCTGTTTAAACTACCGGTAAAAAAAGCACCGACTCGGTGCCAC
TA-U6B/CR7T-gRNA -B	U6B-AgeI-F	CATACCGGTGTTCGACTTGCAGCCTGAAATAC
	CR7T-AgeI-F	CATACCGGTCGTTTTGTCATCGCTTTTTGTCG
	U6B/CR7T-KpnI-R	CATGGTACCAAAAAAAGCACCGACTCGGTGCCAC
TA-U6B/CR7T-gRNA -C	U6B-SpeI-F	CATACTAGT GTTCGACTTGCAGCCTGAAATA
	CR7T-SpeI-F	CATACTAGTCGTTTTGTCATCGCTTTTTGTCG
	U6B/CR7T-SpeI-R	CATACTAGTAAAAAAAGCACCGACTCGGTGCCAC
TA-U6B/CR7T-gRNA -D	U6B-KpnI-F	CATGGTACCGTTCGACTTGCAGCCTGAAATA
	CR7T-KpnI-F	CATGGTACCCGTTTTGTCATCGCTTTTTGTCG
	U6B/CR7T-KpnI-R	CATGGTACCAAAAAAAGCACCGACTCGGTGCCAC

Table S3 List of primers used to construct the transgenic gRNA vector.

Target locus	**Primer name**	**Primer sequence (5' – 3') Forward and Reverse**
yellow	U6B-yw-gRNA1-KOD-F	GGTCGGCTGTGTTTTAGAGCTAGAAATAGCAAGTT
	U6B-yw-gRNA1-KOD-R	ACACTCATCCGAAGTATTGAGGAAAACATACCTA
	CR7T-yw-gRNA1-KOD-F	GGTCGGCTGTGTTTTAGAGCTAGAAATAGCAAGTT
	CR7T-yw-gRNA1-KOD-R	ACACTCATCCGAAAGTCTTCCACTCATATACGCT
	CR7T-yw-gRNA2-KOD-F	ACTGGAACCGGTTTTAGAGCTAGAAATAGCAAGTT
	CR7T-yw-gRNA2-KOD-R	GTCCAAAACCCGAAAGTCTTCCACTCATATACGCT
notch	U6B-notch-gRNA1-KOD-R	CCGTTCTGGCAAAGATGTCCGAAGTATTGAGGAAAACATACCTATA
	U6B-notch-gRNA2-KOD-R	TTGTGTTCGCACGGTGATCCCGAAGTATTGAGGAAAACATACCTATA
	CR7T-notch-gRNA1-KOD-R	CCGTTCTGGCAAAGATGTCCGAAAGTCTTCCACTCATATACGCTA
	CR7T-notch-gRNA2-KOD-R	TTGTGTTCGCACGGTGATCCCGAAAGTCTTCCACTCATATACGCTA
bag of marbles (bam)	U6B-bam-gRNA1-KOD-R	ACTGCTGGTCGTCGTTGCCCGAAGTATTGAGGAAAACATACCTATA
	CR7T-bam-gRNA1-KOD-R	ACTGCTGGTCGTCGTTGCCCGAAAGTCTTCCACTCATATACGCTA
	CR7T-bam-gRNA2-KOD-R	TTAAGGCCAAATGCTCCTCCGAAAGTCTTCCACTCATATACGCTA
	CR7T-bam-gRNA3-KOD-R	CGTACTCACAAGTGGCTTTCCGAAAGTCTTCCACTCATATACGCTA
nanos	U6B-nos-gRNA1-KOD-R	AATACATGTCCTGCAGGCCCGAAGTATTGAGGAAAACATACCTATA
	U6B-nos-gRNA2-KOD-R	CCCATTACGCCGGTGACCCCGAAGTATTGAGGAAAACATACCTATA
	CR7T-nos-gRNA1-KOD-R	AATACATGTCCTGCAGGCCCGAAAGTCTTCCACTCATATACGCTA
	CR7T-nos-gRNA2-KOD-R	CCCATTACGCCGGTGACCCCGAAAGTCTTCCACTCATATACGCTA
cid	U6B-cid-gRNA1-KOD-R	GCTGCCTCCGTCCGGCGTCCGAAGTATTGAGGAAAACATACCTATA
	U6B-cid-gRNA2-KOD-R	GCTGCTCGCGTTTTGCTTTCCGAAGTATTGAGGAAAACATACCTATA
	U6B-cid-gRNA3-KOD-R	AACGACGACGACACGGCCTTC GAAGTATTGAGGAAAACATACCTATA
	U6B-cid-gRNA4-KOD-R	ACTACGGCCTCGAATTCACC GAAGTATTGAGGAAAACATACCTATA
ms(3)k81	U6B-K81-KOD-F<br>U6B-K81-KOD-R	TTACGCGGTAGTTTTAGAGCTAGAAATAGCAAGTT<br>TCAGAAATCCGAAGTATTGAGGAAAACATACCTA
	gRNA-KOD-F	GTTTTAGAGCTAGAAATAGCAAGTT

Table S4 List of primers used for PCR to verify the conditional mutations.

Target locus	Primer name	Primer sequence (5' – 3') Forward and Reverse
yellow	yellow-seq-F	CGGAGCTAATTCCGTATCCA
	yellow-seq-R	CGCCAGGTAGCTCGTATCTC
notch	Notch-seq-F	TGGAAGTGTGACCGTTTTACCC
	Notch-seq-R	GTGGCAAGTTCCATCGTTCAAGCA
bag of marbles (bam)	Bam-Seq-F	CAAAGAGTCTGGACGCCATCAT
	Bam-Seq-R	CGGTTCCACACATTTTCCTTCT
nanos	Nos-Seq-F	TTCGCAGTTGTTTCAAGTTGTCTA
	Nos-Seq-R	ATCTCGTCCGTTTGCTGGTGA
ms(3)k81	K81-seq-F	GAGATTTCTCACTACTGCTCCTCG
	K81-seq-R	ACACGAATTGGATATGCGATAGC
