# Supplementary material for: CRISPR/Cas9 Mediates Efficient Conditional Mutagenesis in Drosophila
Source: G3 (Bethesda). 2014 Sep 5;4(11):2167–73. doi: 10.1534/g3.114.014159 (PMC4232542; doi:10.1534/g3.114.014159)
Supplement: Supporting Information [file supp_g3.114.014159_FigureS1.pdf]

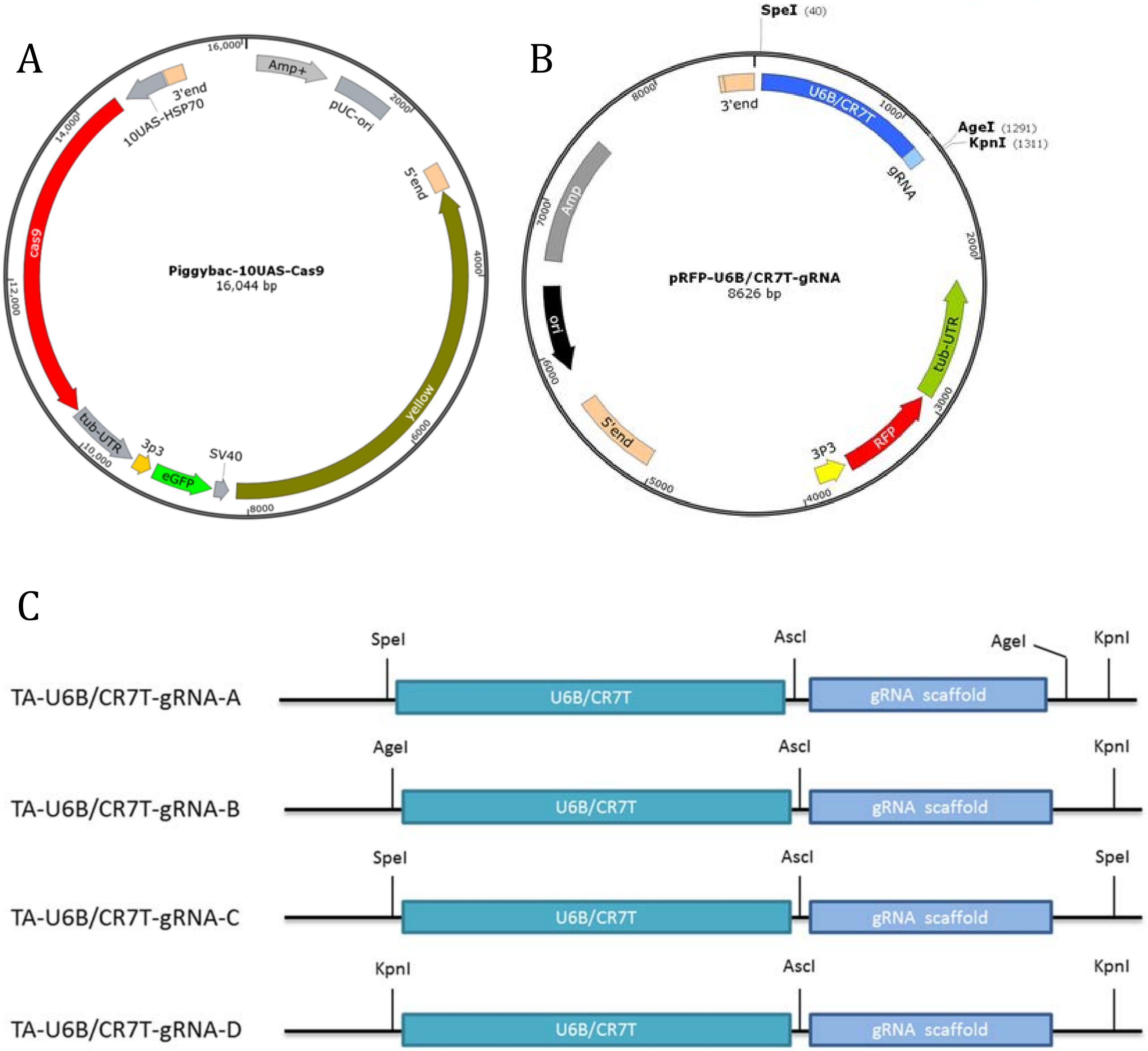
A
Piggybac-10UAS-Cas9
16,044 bp
16,000
2000
4000
6000
8000
10,000
12,000
14,000
Amp+
pUC-ori
5'end
yellow
SV40
eGFP
3p3
tub-UTR
cas9
10UAS-HSP70
3'end
B
SpeI (40)
AgeI (1291)
KpnI (1311)
pRFP-U6B/CR7T-gRNA
8626 bp
1000
2000
3000
4000
5000
6000
7000
8000
U6B/CR7T
gRNA
tub-UTR
RFP
3P3
5'end
ori
Amp
3'end
C
TA-U6B/CR7T-gRNA-A
SpeI
AscI
AgeI
KpnI
U6B/CR7T
gRNA scaffold
TA-U6B/CR7T-gRNA-B
AgeI
AscI
KpnI
U6B/CR7T
gRNA scaffold
TA-U6B/CR7T-gRNA-C
SpeI
AscI
SpeI
U6B/CR7T
gRNA scaffold
TA-U6B/CR7T-gRNA-D
KpnI
AscI
KpnI
U6B/CR7T
gRNA scaffold

D

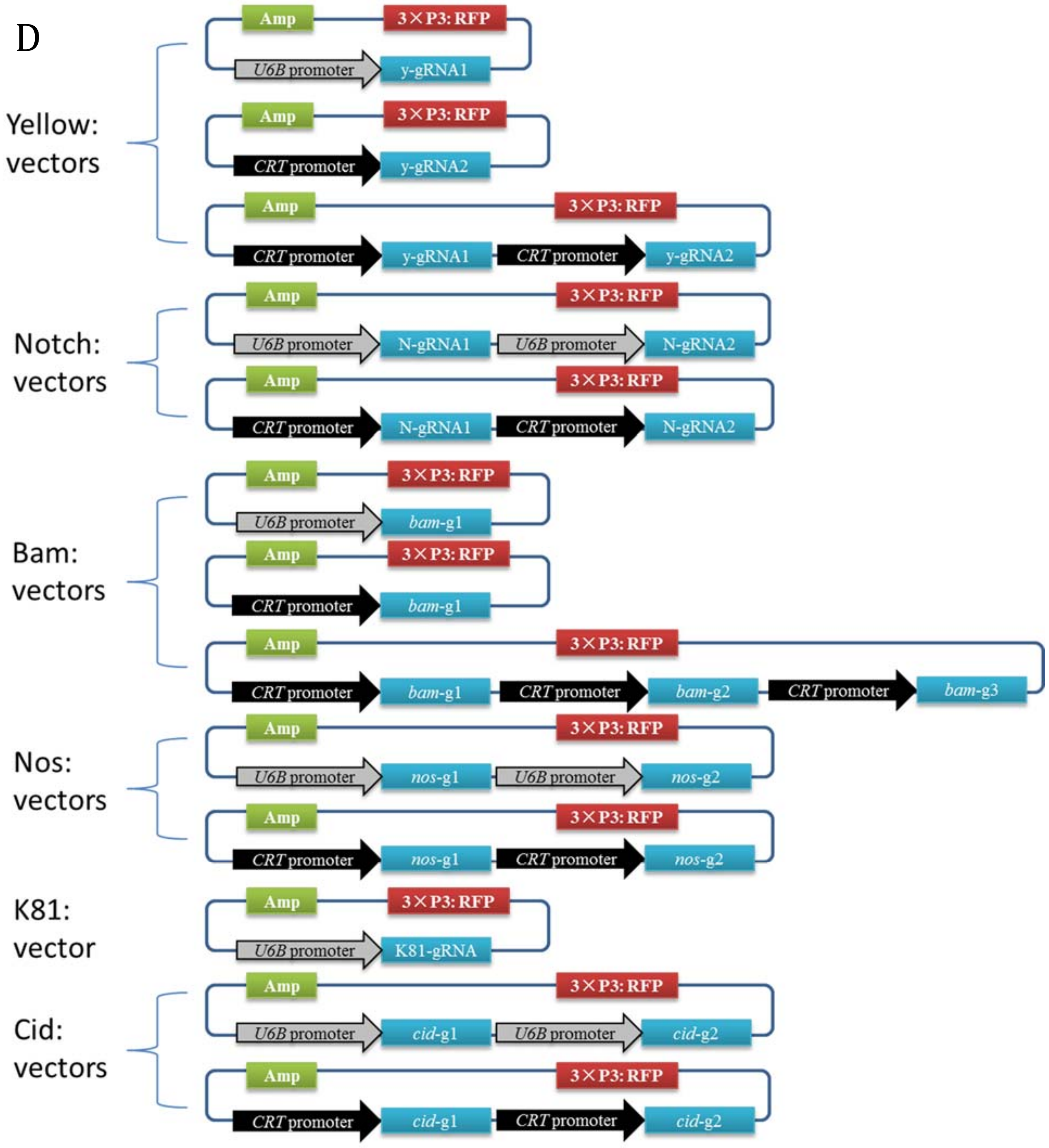

Figure S1 Maps of the plasmids. (A) Piggybac-10UAS-cas9, (B) pRFP-U6B/CR7T-gRNA, and (C) the four backbone plasmids for gRNA insertion. (D) Maps of all transgenic gRNA vectors.
