# Supplementary material for: CRISPR/Cas9 Mediates Efficient Conditional Mutagenesis in Drosophila
Source: G3 (Bethesda). 2014 Sep 5;4(11):2167–73. doi: 10.1534/g3.114.014159 (PMC4232542; doi:10.1534/g3.114.014159)
Supplement: Supporting Information [file supp_g3.114.014159_FigureS2.pdf]

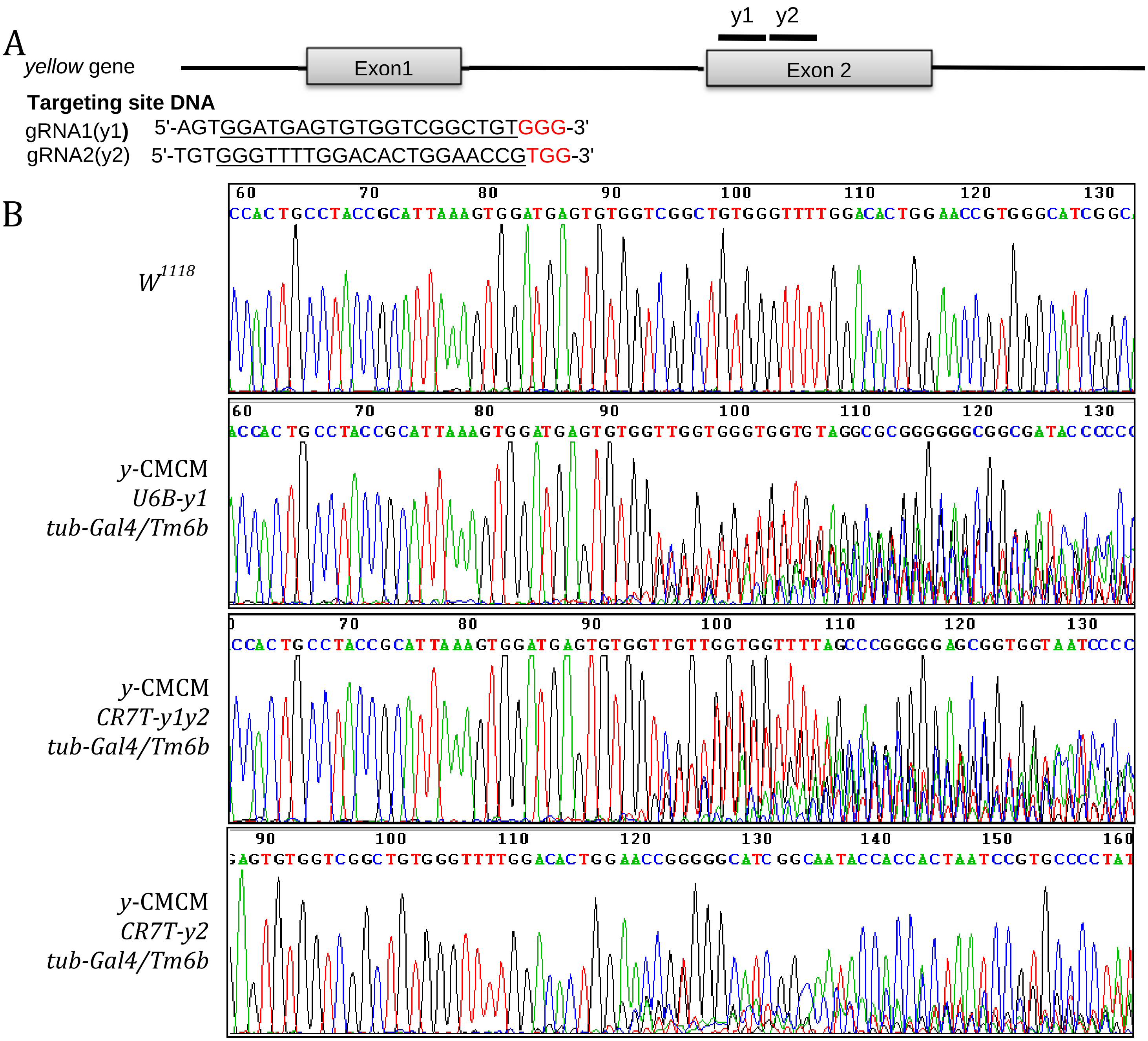

Figure S2 Sequence results for the *y* conditional mutant flies. (A) Sequences and schematic representation of the two gRNAs against the *y* gene. (B) *Tub-Gal4/Tm6b* was used to drive the expression of Cas9 in whole bodies, and three vectors, *U6B-y1*, *CR7T-y2*, and *CR7T-y1y2,* were used to drive the expression of gRNA. The mutations were induced exactly at the target locus. *w*[1118] was used as the control.
