# Supplementary material for: CRISPR/Cas9 Mediates Efficient Conditional Mutagenesis in Drosophila
Source: G3 (Bethesda). 2014 Sep 5;4(11):2167–73. doi: 10.1534/g3.114.014159 (PMC4232542; doi:10.1534/g3.114.014159)
Supplement: Supporting Information [file supp_g3.114.014159_FigureS3.pdf]

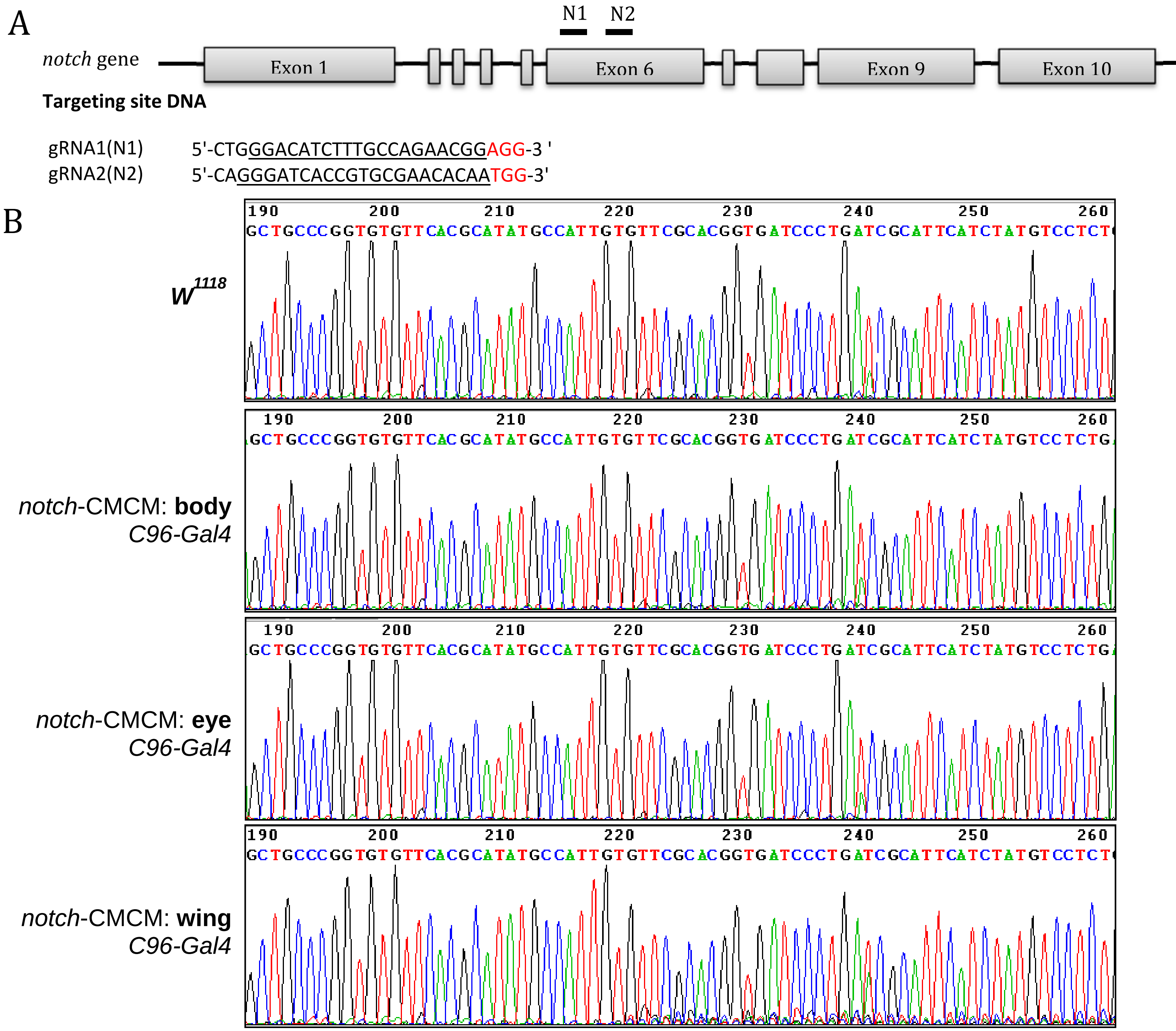

Figure S3 Sequence results for *notch* conditional mutant flies. (A) Sequences and schematic representation of two gRNAs against the *notch* gene. (B) *C96-Gal4* was used to drive the expression of Cas9 specifically in the blade region of the wing imaginal disc. The body, eye, and wing from the *notch* conditional mutant flies were sequenced. The mutation was induced only in the wing tissue at the target locus. w^{1118} was used as the control.
