# Supplementary material for: CRISPR/Cas9 Mediates Efficient Conditional Mutagenesis in Drosophila
Source: G3 (Bethesda). 2014 Sep 5;4(11):2167–73. doi: 10.1534/g3.114.014159 (PMC4232542; doi:10.1534/g3.114.014159)
Supplement: Supporting Information [file supp_g3.114.014159_FigureS4.pdf]

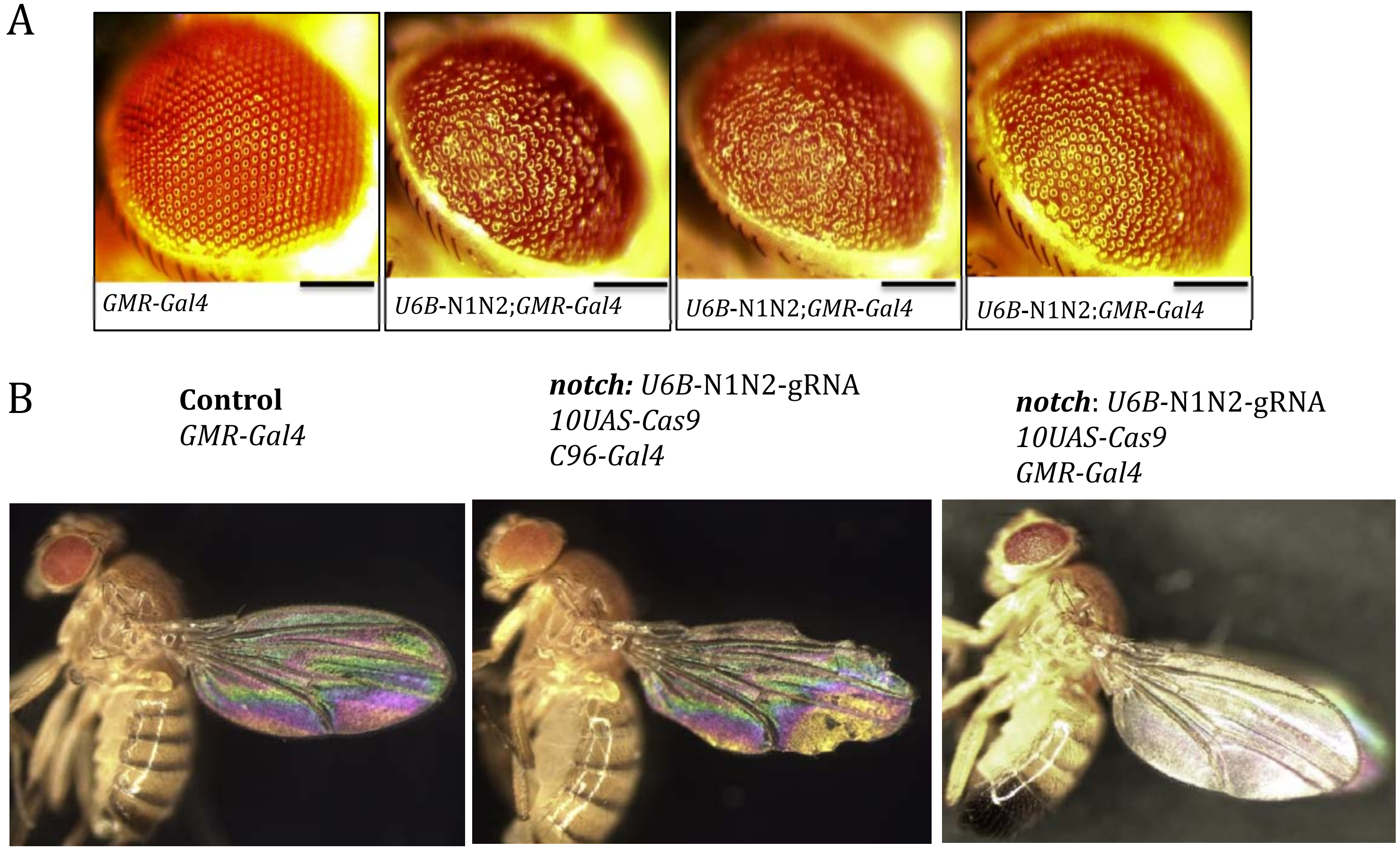

Figure S4 Phenotypes resulting from the conditional *notch* mutation in the eye and wing. (A) The conditional mutant flies showed rougher eyes than the *GMR-Gal4* control. Scale bars: 100 µm. (B) The whole-fly images for *notch* conditional mutagenesis are shown. The left-hand image used the *GMR-Gal4* fly as a control, the middle image is from the *notch* conditional mutagenesis driven by wing-specific *C96-Gal4*, and the right-hand image is from the *notch* conditional mutagenesis driven by eye-specific *GMR-Gal4*. *U6B-N1N1* was used to drive the expression of gRNA.
