# Supplementary material for: CRISPR/Cas9 Mediates Efficient Conditional Mutagenesis in Drosophila
Source: G3 (Bethesda). 2014 Sep 5;4(11):2167–73. doi: 10.1534/g3.114.014159 (PMC4232542; doi:10.1534/g3.114.014159)
Supplement: Supporting Information [file supp_g3.114.014159_FigureS5.pdf]

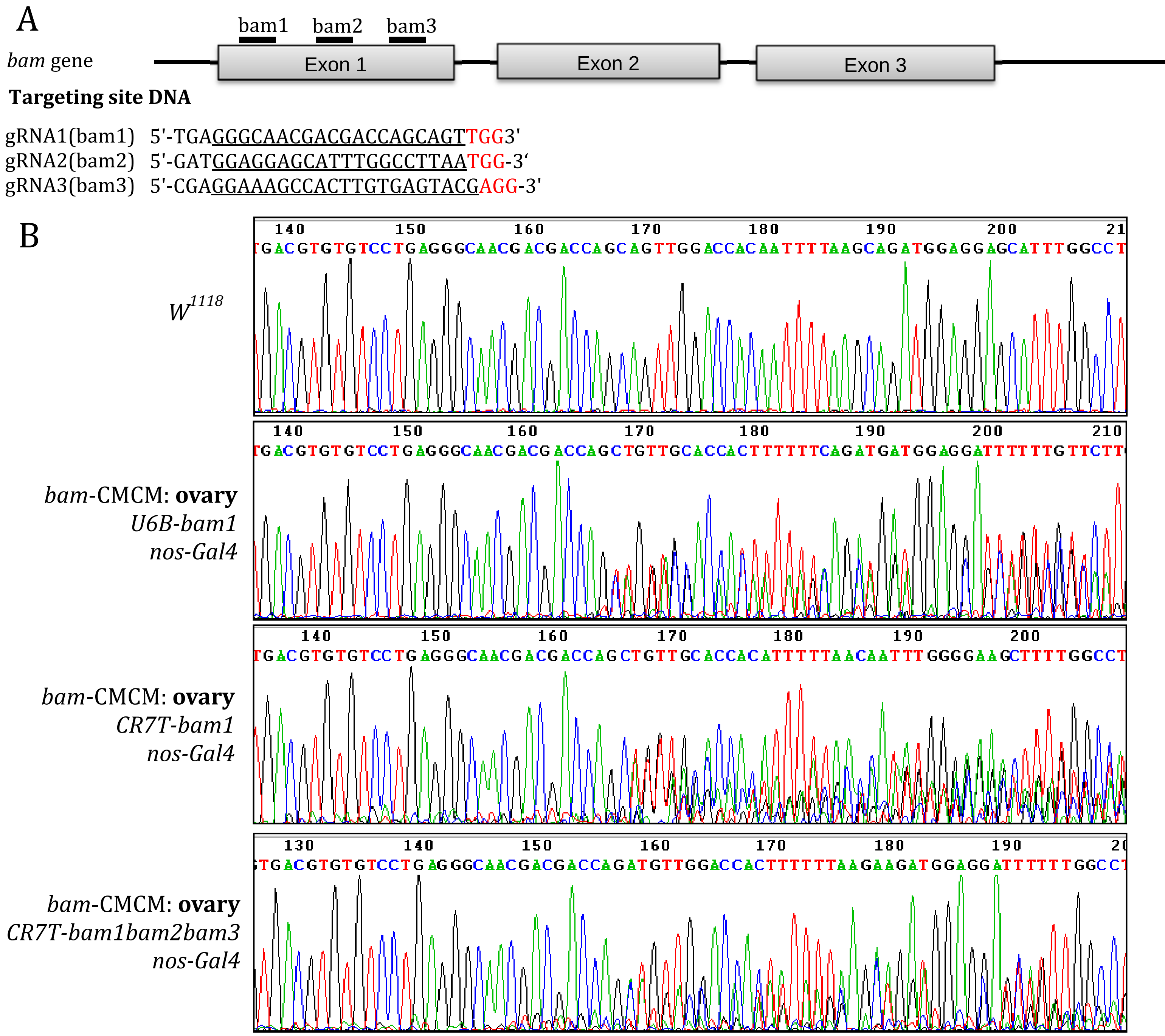

Figure S5 Sequence results for the ovaries of *bam* conditional mutant flies. (a) The sequences and a schematic representation of three gRNAs against the *bam* gene are shown. (b) *Nos-Gal4* was used to drive the expression of Cas9 specifically in the ovary, and three vectors, *U6B-bam1*, *CR7T-bam2,* and *CR7T-bam1bam2bam3*, were used to drive the expression of gRNA. The mutations were induced exactly at the target locus. A w^{1118} fly was used as the control.
