# Supplementary material for: CRISPR/Cas9 Mediates Efficient Conditional Mutagenesis in Drosophila
Source: G3 (Bethesda). 2014 Sep 5;4(11):2167–73. doi: 10.1534/g3.114.014159 (PMC4232542; doi:10.1534/g3.114.014159)
Supplement: Supporting Information [file supp_g3.114.014159_FigureS7.pdf]

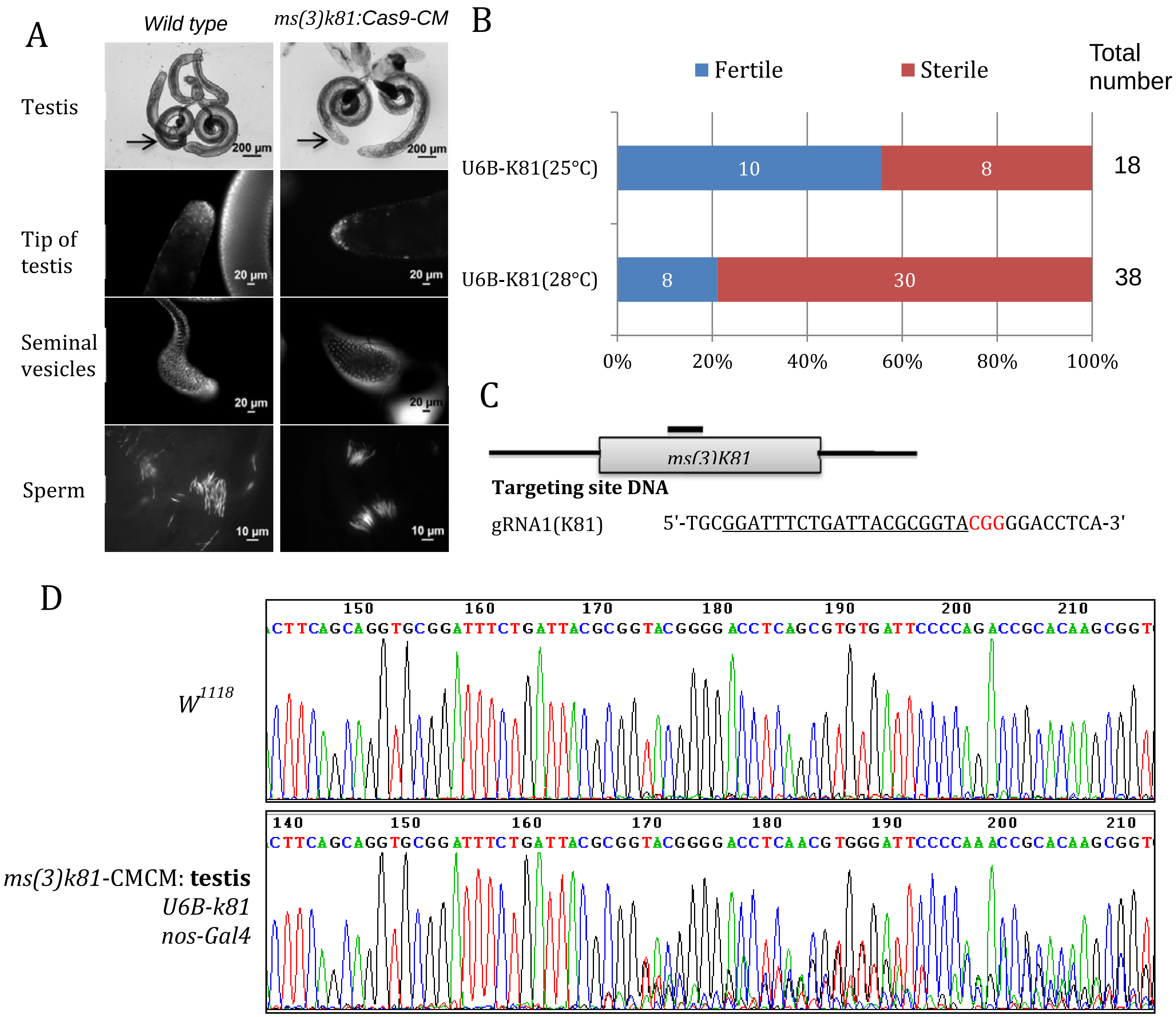

Figure S7 Conditional mutation of the *ms(3)k81* gene via the Cas9-mediated conditional mutagenesis (CMCM) system. (A) From the top to bottom of each column: whole testis (light), tip of the testis (DAPI), seminal vesicles (DAPI), and sperm detection (DAPI). *Nos-Gal4* was used to drive the expression of Cas9, and *U6B-K81* was used to drive the expression of gRNA. *ms(3)k81:Cas9-CM* was the testis from conditional mutant fly via the CMCM system. (B) Results of a fertility test are shown for flies for the CMCM system using the *ms(3)k81* gene. (C, D) The sequences and

a schematic representation of gRNAs against the *ms(3)k81* gene. Sequence results for the testes from the *ms(3)k81* conditional mutant flies. The mutations induced by CMCM are located at the targeted locus.
