# Supplementary material for: CRISPR/Cas9 Mediates Efficient Conditional Mutagenesis in Drosophila
Source: G3 (Bethesda). 2014 Sep 5;4(11):2167–73. doi: 10.1534/g3.114.014159 (PMC4232542; doi:10.1534/g3.114.014159)
Supplement: Supporting Information [file supp_g3.114.014159_TableS1.pdf]

Table S1 Sites targeted for each *Drosophila* gene and pRFP-gRNA constructions for the target loci.

Target gene		Target site (5' to 3') (PAM is underlined)	pRFP-gRNA	Number of gRNA
yellow	y1	GGATGAGTGTGGTCGGCTGTGGG	pRFP-U6B-y1-gRNA	1
	y2	GGGTTTTGGACACTGGAACCGTGG	pRFP-CR7T-y2-gRNA	1
			pRFP-CR7T-y1y2-gRNA	2
notch	N1	GGACATCTTTGCCAGAACGGAGG	pRFP-U6B-N1N2-gRNA	2
	N2	GGGATCACCGTGCGAACACAATGG	pRFP-CRT-N1N2-gRNA	2
bag of marbles (bam)	bam1	GGGCAACGACGACCAGCAGTTGG	pRFP-U6B-bam1-gRNA	1
	bam2	GGAGGAGCATTTGGCCTTAATGG	pRFP-CR7T-bam1-gRNA	1
	bam3	GGAAAGCCACTTGTGAGTACGAGG	pRFP-CR7T-bam1bam2bam3-gRNA	3
nanos	nos1	GGGCCTGCAGGACATGTATTTGG	pRFP-U6B-nos1nos2-gRNA	2
	nos2	GGGGTCACCGGCGTAATGGGCGG	pRFP-CR7T-nos1nos2-gRNA	2
cid	cid1	GGACGCCGGACGGAGGCAGCCGG	pRFP-U6B-cid1cid2-gRNA	2
	cid2	GGAAAGCAAAACGCGAGCAGCAGG	pRFP-CR7T-cid1cid2-gRNA	2
ms(3)K81	K81	GGATTTCTGATTACGCGGTACGG	pRFP-U6B-K81-gRNA	1
