# Supplementary material for: CRISPR/Cas9 Mediates Efficient Conditional Mutagenesis in Drosophila
Source: G3 (Bethesda). 2014 Sep 5;4(11):2167–73. doi: 10.1534/g3.114.014159 (PMC4232542; doi:10.1534/g3.114.014159)
Supplement: Supporting Information [file supp_g3.114.014159_TableS2.pdf]

Table S2 List of primers used to construct the 10UAS-Cas9/TA-gRNA vector.

Plasmid	Primer name	Primer sequence (5' – 3') Forward and Reverse
piggyBac-10UAS-cas9	10UAS-XmaI-F	CCCGGGCTCGATCCGCTTGCATGC
	10UAS-NotI-R	GCGGCCGCCAATTCCCTATTCAGAGTTCTCTTCTT
	αTub84B 3'UTR-XhoI-F	CTACTACTACTCGAGCGGCCATCGAATTCGAGCTC
	αTub84B 3'UTR-SpeISalI-R	CATCATCATGTCGACACTAGTTAGAGAGCTTCGCATGGTTTTGCC
TA-U6B/CR7T-gRNA -A	U6B-NotIsphIspeIFseI-F	GCGGCCGCATGCACTAGTGGCCGGCCGTTCGACTTGCAGCCTGAAATAC
	CR7T-XhoIspeIFseI-F	CTCGAGACTAGTGGCCGGCCCGTTTTGTCATCGCTTTTTGTCG
	U6B/CR7T-AgeI/KpnI-R	GGTACCTGTTTAAACTACCGGTAAAAAAAGCACCGACTCGGTGCCAC
TA-U6B/CR7T-gRNA -B	U6B-AgeI-F	CATACCGGTGTTCGACTTGCAGCCTGAAATAC
	CR7T-AgeI-F	CATACCGGTCGTTTTGTCATCGCTTTTTGTCG
	U6B/CR7T-KpnI-R	CATGGTACCAAAAAAAGCACCGACTCGGTGCCAC
TA-U6B/CR7T-gRNA -C	U6B-SpeI-F	CATACTAGT GTTCGACTTGCAGCCTGAAATA
	CR7T-SpeI-F	CATACTAGTCGTTTTGTCATCGCTTTTTGTCG
	U6B/CR7T-SpeI-R	CATACTAGTAAAAAAAGCACCGACTCGGTGCCAC
TA-U6B/CR7T-gRNA -D	U6B-KpnI-F	CATGGTACCGTTCGACTTGCAGCCTGAAATA
	CR7T-KpnI-F	CATGGTACCCGTTTTGTCATCGCTTTTTGTCG
	U6B/CR7T-KpnI-R	CATGGTACCAAAAAAAGCACCGACTCGGTGCCAC
