# Supplementary material for: CRISPR/Cas9 Mediates Efficient Conditional Mutagenesis in Drosophila
Source: G3 (Bethesda). 2014 Sep 5;4(11):2167–73. doi: 10.1534/g3.114.014159 (PMC4232542; doi:10.1534/g3.114.014159)
Supplement: Supporting Information [file supp_g3.114.014159_TableS3.pdf]

Table S3 List of primers used to construct the transgenic gRNA vector.

Target locus	**Primer name**	**Primer sequence (5' – 3') Forward and Reverse**
yellow	U6B-yw-gRNA1-KOD-F	GGTCGGCTGTGTTTTAGAGCTAGAAATAGCAAGTT
	U6B-yw-gRNA1-KOD-R	ACACTCATCCGAAGTATTGAGGAAAACATACCTA
	CR7T-yw-gRNA1-KOD-F	GGTCGGCTGTGTTTTAGAGCTAGAAATAGCAAGTT
	CR7T-yw-gRNA1-KOD-R	ACACTCATCCGAAAGTCTTCCACTCATATACGCT
	CR7T-yw-gRNA2-KOD-F	ACTGGAACCGGTTTTAGAGCTAGAAATAGCAAGTT
	CR7T-yw-gRNA2-KOD-R	GTCCAAAACCCGAAAGTCTTCCACTCATATACGCT
notch	U6B-notch-gRNA1-KOD-R	CCGTTCTGGCAAAGATGTCCGAAGTATTGAGGAAAACATACCTATA
	U6B-notch-gRNA2-KOD-R	TTGTGTTCGCACGGTGATCCCGAAGTATTGAGGAAAACATACCTATA
	CR7T-notch-gRNA1-KOD-R	CCGTTCTGGCAAAGATGTCCGAAAGTCTTCCACTCATATACGCTA
	CR7T-notch-gRNA2-KOD-R	TTGTGTTCGCACGGTGATCCCGAAAGTCTTCCACTCATATACGCTA
bag of marbles (bam)	U6B-bam-gRNA1-KOD-R	ACTGCTGGTCGTCGTTGCCCGAAGTATTGAGGAAAACATACCTATA
	CR7T-bam-gRNA1-KOD-R	ACTGCTGGTCGTCGTTGCCCGAAAGTCTTCCACTCATATACGCTA
	CR7T-bam-gRNA2-KOD-R	TTAAGGCCAAATGCTCCTCCGAAAGTCTTCCACTCATATACGCTA
	CR7T-bam-gRNA3-KOD-R	CGTACTCACAAGTGGCTTTCCGAAAGTCTTCCACTCATATACGCTA
nanos	U6B-nos-gRNA1-KOD-R	AATACATGTCCTGCAGGCCCGAAGTATTGAGGAAAACATACCTATA
	U6B-nos-gRNA2-KOD-R	CCCATTACGCCGGTGACCCCGAAGTATTGAGGAAAACATACCTATA
	CR7T-nos-gRNA1-KOD-R	AATACATGTCCTGCAGGCCCGAAAGTCTTCCACTCATATACGCTA
	CR7T-nos-gRNA2-KOD-R	CCCATTACGCCGGTGACCCCGAAAGTCTTCCACTCATATACGCTA
cid	U6B-cid-gRNA1-KOD-R	GCTGCCTCCGTCCGGCGTCCGAAGTATTGAGGAAAACATACCTATA
	U6B-cid-gRNA2-KOD-R	GCTGCTCGCGTTTTGCTTTCCGAAGTATTGAGGAAAACATACCTATA
	U6B-cid-gRNA3-KOD-R	AACGACGACGACACGGCCTTC GAAGTATTGAGGAAAACATACCTATA
	U6B-cid-gRNA4-KOD-R	ACTACGGCCTCGAATTCACC GAAGTATTGAGGAAAACATACCTATA
ms(3)k81	U6B-K81-KOD-F<br>U6B-K81-KOD-R	TTACGCGGTAGTTTTAGAGCTAGAAATAGCAAGTT<br>TCAGAAATCCGAAGTATTGAGGAAAACATACCTA
	gRNA-KOD-F	GTTTTAGAGCTAGAAATAGCAAGTT
