# Supplementary material for: CRISPR/Cas9 Mediates Efficient Conditional Mutagenesis in Drosophila
Source: G3 (Bethesda). 2014 Sep 5;4(11):2167–73. doi: 10.1534/g3.114.014159 (PMC4232542; doi:10.1534/g3.114.014159)
Supplement: Supporting Information [file supp_g3.114.014159_TableS4.pdf]

Table S4 List of primers used for PCR to verify the conditional mutations.

Target locus	Primer name	Primer sequence (5' – 3') Forward and Reverse
yellow	yellow-seq-F	CGGAGCTAATTCCGTATCCA
	yellow-seq-R	CGCCAGGTAGCTCGTATCTC
notch	Notch-seq-F	TGGAAGTGTGACCGTTTTACCC
	Notch-seq-R	GTGGCAAGTTCCATCGTTCAAGCA
bag of marbles (bam)	Bam-Seq-F	CAAAGAGTCTGGACGCCATCAT
	Bam-Seq-R	CGGTTCCACACATTTTCCTTCT
nanos	Nos-Seq-F	TTCGCAGTTGTTTCAAGTTGTCTA
	Nos-Seq-R	ATCTCGTCCGTTTGCTGGTGA
ms(3)k81	K81-seq-F	GAGATTTCTCACTACTGCTCCTCG
	K81-seq-R	ACACGAATTGGATATGCGATAGC
